# Recent Advances and Challenges towards Sustainable Polyhydroxyalkanoate (PHA) Production

**DOI:** 10.3390/bioengineering4020055

**Published:** 2017-06-11

**Authors:** Constantina Kourmentza, Jersson Plácido, Nikolaos Venetsaneas, Anna Burniol-Figols, Cristiano Varrone, Hariklia N. Gavala, Maria A. M. Reis

**Affiliations:** 1 UCIBIO-REQUIMTE, Department of Chemistry, Faculdade de Ciências e Tecnologia/Universidade Nova de Lisboa, 2829-516 Caparica, Portugal; amr@fct.unl.pt; 2 Centre for Cytochrome P450 Biodiversity, Institute of Life Science, Swansea University Medical School, Singleton Park, Swansea SA2 8PP, UK; j.e.placidoescobar@swansea.ac.uk; 3 Faculty of Engineering and the Environment, University of Southampton, Highfield, Southampton SO17 1BJ, UK; n.venetsaneas@aston.ac.uk; 4 European Bioenergy Research Institute (EBRI), Aston University, Aston Triangle, Birmingham B4 7ET, UK; 5 Department of Chemical and Biochemical Engineering, Center for Bioprocess Engineering, Søltofts Plads, Technical University of Denmark, Building 229, 2800 Kgs. Lyngby, Denmark; afig@kt.dtu.dk (A.B.-F.); cvar@kt.dtu.dk (C.V.); hnga@kt.dtu.dk (H.N.G.)

**Keywords:** polyhydroxyalkanoates, biopolymers, renewable feedstock, mixed microbial consortia, enrichment strategy, pure cultures, synthetic biology, downstream processing

## Abstract

Sustainable biofuels, biomaterials, and fine chemicals production is a critical matter that research teams around the globe are focusing on nowadays. Polyhydroxyalkanoates represent one of the biomaterials of the future due to their physicochemical properties, biodegradability, and biocompatibility. Designing efficient and economic bioprocesses, combined with the respective social and environmental benefits, has brought together scientists from different backgrounds highlighting the multidisciplinary character of such a venture. In the current review, challenges and opportunities regarding polyhydroxyalkanoate production are presented and discussed, covering key steps of their overall production process by applying pure and mixed culture biotechnology, from raw bioprocess development to downstream processing.

## 1. Introduction

Polyhydroxyalkanoates (PHAs) are a class of renewable, biodegradable, and bio-based polymers, in the form of polyesters. Together with polylactic acid (PLA) and polybutylene succinate (PBS), they are considered the green polymers of the future since they are expected to gradually substitute conventional plastics with similar physicochemical, thermal, and mechanical properties such as polypropylene (PP) and low-density polyethylene (LDPE) [1,2]. While PLA and PBS are produced upon polymerization of lactic and succinic acid respectively, PHA polymerization is performed naturally by bacteria.

A wide variety of bacteria are able to accumulate PHAs in the form of intracellular granules, as carbon and energy reserves. PHA accumulation is usually promoted when an essential nutrient for growth is present in limited amount in the cultivation medium, whereas carbon is in excess. Although, several bacteria are able to produce PHAs during growth and do not require growth-limiting conditions. This carbon storage is used by bacteria as an alternate source of fatty acids, metabolized under stress conditions, and is the key mechanism for their survival [3]. Up to 150 different PHA structures have been identified so far [4]. In general, PHAs are classified into two groups according to the carbon atoms that comprise their monomeric unit. Short-chain-length PHAs (scl-PHAs) consist of 3–5 carbon atoms, whereas medium-chain-length PHAs (mcl-PHAs) consist of 6–14 carbon atoms. PHB, the most well-known scl-PHA member, is characterized as a stiff and brittle material and is difficult to be processed due to its crystalline nature. The incorporation of 3-hydroxyvalerate (HV) units in PHB, results in the production of the copolymer poly(3-hydroxybutyrate-co-3-hydroxyvalerate), or else PHBV. PHBV is a material that becomes tougher, more flexible, and broader to thermal processing when its molar fraction in the copolymer increases [5]. scl-PHAs are mostly used for the production of disposable items and food packaging materials. On the other hand, mcl-PHAs are characterized as elastomers and they are suitable for high value-added application, such as surgical sutures, implants, biodegradable matrices for drug delivery, etc. [4].

Taking into account the recalcitrance of conventional plastics in the environment, replacement of synthetic plastics with PHAs would have huge benefits for the society and the environment [6]. Wide commercialization and industrialization of PHAs is still struggling due to their high production cost, resulting in higher prices compared to conventional polymers. While the price of polymers such as PP and PE is around US$0.60–0.87/lb, PHA biopolymer cost is estimated to be 3–4 times higher, ranging between US$2.25–2.75/lb [7,8]. Although several companies have initiated and industrialized the production of PHAs, as presented in Table 1, there are still major issues that need to be addressed in an effort to reduce the overall production cost. The main reasons for their high cost is the high price of high purity substrates, such as glucose, production in discontinuous batch and fed-batch cultivation modes, and large amount of solvents and/or labor regarding their downstream processing. The increasing availability of raw renewable materials and increasing demand and use of biodegradable polymers for bio-medical, packaging, and food applications along with favorable green procurement policies are expected to benefit PHA market growth. According to a recent report, published in 2017, the global PHA market is expected to reach US$93.5 million by 2021, from an estimated US$73.6 million within 2016, characterized by a compound annual growth rate (CAGR) of 4.88% [9].

In the following sections, the advantages and drawbacks of PHA production employing both pure and mixed culture biotechnology are presented and discussed, as well as several approaches regarding their downstream processing in order to identify bottlenecks and opportunities to leverage PHA production.

## 2. PHA Production by Pure Bacterial Cultures

Pure culture biotechnology is implemented on an industrial scale, since a wide variety of food, pharmaceutical, and cosmetic agents derive as metabolic compounds from certain bacterial strains. Within the last few decades, research has been focused on finding ways to decrease the high production cost of PHAs. One of the main contributors to their high cost is the use of high purity substrates, which can account for 45% of the total production cost [10]. Instead, renewable feedstocks are being explored and researchers have been developing bioprocesses for the valorization of waste streams and by-products. In addition, current legislation and policies promote biodegradable waste management solutions other than disposal in landfills. Since every type of waste stream or by-product has different composition, selecting the appropriate biocatalyst is of great importance. In cases where the raw material is rich in carbon and nutrients, a growth-associated PHA producer would be selected, such as *Alcaligenes latus* or *Paracoccus denitrificans*. Conversely, in cases where the feedstock lacks an essential nutrient for growth such as nitrogen, phosphorous, etc., PHA accumulation using non-growth-associated bacteria would be ideal, i.e., *Cupriavidus necator*.

Apart from well-known species involved in industrial PHA production such as *Alcaligenes latus*, *Cupriavidus necator*, and *Pseudomonas putida*, bacteria need to combine several features in order be selected and regarded as promising PHA producers. Such features include their performance utilizing renewable feedstocks and/or environmental pollutants, seawater instead of fresh water, possibility of PHA production under open, non-sterile conditions, and their potential to develop contamination-free continuous bioprocesses. The use of agricultural byproducts and forest residues as an abundant and renewable source of lignocellulosic material for PHA production, is mainly considered after its physicochemical or biological hydrolysis. However, a few microorganisms possess the ability to saccharify cellulose and simultaneously produce PHAs. Moreover, PHA producers isolated from contaminated sites may be regarded for combined PHA production and bioremediation of toxic pollutants and post-consumer plastics. In addition, microorganisms isolated from hypersaline environments are considered the most promising ones, since they combine several benefits with a huge potential for reducing PHA production cost, namely in the downstream step. Last but not least, within recent years synthetic biology tools are continuously being developed in order to provide solutions to industrial challenges such as maximizing cellular capacity to ‘make more space’ for PHA accumulation, manipulating PHA composition to design polymers for high value-added applications and enhancing PHA efficiency.

### 2.1. Lignocellulose Degraders

#### 2.1.1. Saccharophagus Degradans

*Saccharophagus degradans*, formerly known as *Microbulbifer degradans*, refers to a species of marine bacteria capable of degrading complex marine polysaccharides, such as cellulose of algal origin and higher plant material [11,12,13]. So far, the only strain reported is *S. degradans* 2-40, that was isolated from decaying marsh cord grass *Spartina alterniflora*, in the Chesapeake Bay watershed [14]. It is a Gram-negative, aerobic, rod-shaped, and motile γ-proteobacterium and is able to use a variety of different complex polysaccharides as its sole carbon and energy source, including agar, alginate, cellulose, chitin, β-glucan, laminarin, pectin, pullulan, starch, and xylan [11,13,15,16,17].

The key enzymes involved in PHA biosynthesis—β-ketothiolase, acetoacetyl-CoA reductase, and PHA synthase—have been identified in the genome of *S. degradans* 2-40 [12,18]. Preliminary studies have been performed in order to evaluate the feasibility of *S. degradans* to produce PHAs from d-glucose and d-cellobiose as the sole carbon source in minimal media comprised of sea salts [19]. In addition, the authors evaluated the capability of the strain to degrade lignocellulosic material in the form of tequila bagasse (*A. tequilana*). According to the results obtained, it was shown that *S. degradans* successfully degraded and utilized cellulose as the primary carbon source to grow and produce PHB. However, PHB yields were not reported, so as to evaluate the efficiency of the process, but it became evident that prior hydrolysis of the lignocellulosic material is not required. This is considered positive since it can contribute to up-stream processing cost reduction and thus encourage further research employing the certain strain. In another study, Gonzalez-Garcia et al. [20], investigated PHA production using glucose as the sole carbon source and a culture medium designed according to bacterial biomass and seawater composition. Experiments were performed using a two-step batch strategy, where in the first step bacterial growth was performed under balanced conditions for 24 h, whereas in the second step cells were aseptically transferred to a fresh nitrogen deficient medium and incubated for 48 h. Under these conditions PHA content reached up to 17.2 ± 2.7% of the cell dry weight (CDW). 

PHB biosynthesis from raw starch in fed-batch mode was also investigated and the results were compared to the ones obtained using glucose as the carbon source under the same conditions [21]. When starch was used PHA yield, content, and productivity reached up to 0.14 ± 0.02 g/g, 17.5 ± 2.7% of CDW and 0.06 ± 0.01 g/L h, respectively. In the case where glucose was fed the respective values were higher but still low compared to other PHA producers. However, only a few microorganisms have been reported to directly utilize raw starch for PHA production [21,22]. During the experiments, the authors observed the simultaneous production of organic acids and exopolymers and this was the main reason for the low PHB accumulation. Higher PHA efficiency could be achieved by optimizing cultivation parameters to drive carbon flux towards PHA biosynthesis and also by applying genetic engineering to knock out genes responsible for the production of side products such as exopolymers.

PHA production in aquarium salt medium supplemented with 1% of different types of cellulosic substrates such as α-cellulose, avicel PH101, sigmacell 101, carboxymethyl cellulose (CMC), and cellobiose have also been studied [23]. In flask experiments, PHB production was 11.8, 14.6, 13.7, and 12.8% of the DCW respectively. Fed-batch cultivation strategy resulted in increased PHB contents reaching up to 52.8% and 19.2% of the DCW using glucose and avicel respectively, as carbon sources.

Recently, another approach towards PHA biosynthesis from *S. degradans* was proposed. During their experiments, Sawant et al. [24] observed that *Bacillus cereus* (KF801505) was growing together with *S. degradans* 2-40 as a contaminant and had the ability of producing high amounts of PHAs [25]. In addition, the viability and agar degradation potential of *S. degradans* increased with the presence of *B. cereus*. Taking those into account, they further investigated the ability of co-cultures of *S. degradans* and *B. cereus* to produce PHAs using 2% w/v agarose and xylan without any prior treatment. PHA contents obtained from agarose and xylan were 19.7% and 34.5% of the DCW respectively when co-cultures were used compared to 18.1% and 22.7% achieved by pure cultures of *S. degradans*. This study reported for the first time the production of PHAs using agarose. Moreover, according to the results, the highest PHA content from xylan was obtained using a natural isolate. So far, only recombinant *Escherichia coli* has been reported to produce 1.1% PHA from xylan, which increased to 30.3% and 40.4% upon supplementation of arabinose and xylose, respectively [26].

These unique features of *S. degradans* open up the possibility to use it as a source of carbohydrases in order to saccharify lignocellulosic materials. Thus, coupled hydrolysis and fermentation is a promising alternative for the production of PHAs using carbon sources that may derive from biomass residues of different origin (Table 2). However, saccharification and coupled PHA production need to be studied in detail in order to understand their potential and find ways to increase the rates of their processes.

#### 2.1.2. Caldimonas Taiwanensis

*Caldimonas taiwanensis* is a bacterial strain isolated from hot spring water in southern Taiwan in 2004 [27]. Researchers had been searching for thermophilic amylase-producing bacteria since those enzymes are of high industrial importance for the food and pharmaceutical sector. In addition, since starch hydrolysis is known to be faster at relatively high temperatures, thermophilic amylases are usually preferred [28]. Upon morphological and physiological characterization, it was shown that this Gram-negative, aerobic, rod shaped bacteria can form PHB granules.

A few years later, Sheu et al. [22] investigated PHA production from a wide variety of carbon sources. At first cultivation of *C. taiwanensis* on a three-fold diluted Luria-Bertani medium supplemented with sodium gluconate, fructose, maltose, and glycerol as the sole carbon sources under optimal nitrogen limiting conditions, C/N = 30 was performed. PHB contents reached up to 70, 62, 60, and 52% of the CDW at 55 °C in shake flask experiments. In the next step, fatty acids were tested as sole carbon sources for growth and PHA production. It was observed that the strain did not grow at a temperature between 45 °C or 55 °C while no PHA was formed. Nevertheless, when mixtures of gluconate and valerate were provided bacterial growth was feasible and PHA cellular content reached up to 51% of its CDW. The presence of valerate induced the presence of HV units in the polymer resulting in the production of a PHBV copolymer constituting of 10–95 mol % HV depending on the relative valerate concentration in the mixture. Moreover, mixtures of commercially available starches and valerate were evaluated for PHA production at 50 °C. The carbon source mixture consisted of 1.5% starch and 0.05% valerate. Starch types examined were cassava, corn, potato, sweet potato, and wheat starch. After 32 h of cultivation PHBV copolymer was produced in all cases, composed of approximately 10 mol % HV. PHA contents of 67, 65, 55, 52, and 42% of its CDW were achieved respectively.

Despite the fact that biotechnological process using thermophilic bacteria need to be performed at high temperatures, they reduce the risk of contamination. Another advantage is the fact that thermophiles grow faster compared to mesophiles, therefore less time is needed to achieve maximum PHA accumulation [22]. Moreover, employing *C. taiwanensis* for PHA production using starch-based raw materials is extremely beneficial, in economic terms, since no prior saccharification is required. On the other hand, as mentioned before, *C. taiwanensis* cannot grow on fatty acids but when a mixture of valerate and gluconate/or starch is supplied bacterial growth and PHA accumulation occurs in the form of PHBV copolymer. However, the concentration of fatty acids may result in toxicity for bacterial cells, that up to a point can be overcome by the fast growth of cells. In addition, since amylose, amylopectin, and nitrogen contents vary between types of starch, prior characterization needs to be performed. The feasibility of enzymatic degradation of amylose and amylopectin is considered a key factor as it regulates the amount of sugars present in the medium. Last but not least, nitrogen content should be also controlled as high amounts favor biomass growth instead of PHA accumulation.

### 2.2. Bioremediation Technologies Allowing PHA Production

One of the major causes of environmental pollution is the presence of volatile aromatic hydrocarbons such as benzene, toluene, ethylbenzene, and xylene (BTEX) that are found in crude oil and petroleum products. In addition, huge amounts of starting materials for the production of petrochemical based plastics, such as styrene, are released annually [29]. Moreover, chemical additives in plastics, which are accumulated in the environment due to their recalcitrance, can leach out and are detectable in aquatic environments, dust and air because of their high volatility [30]. Also, textile dyes and effluents are one of the worst polluters of our precious water bodies and soils. All the above are posing mutagenic, carcinogenic, allergic, and cytotoxic threats to all life forms [31].

Since PHAs are known to have a functional role in bacterial survival under stress conditions, toxic environments characterized by poor nutrient availability are proven to be important sources of PHA producers [32]. Several attempts have been made within the last decade to explore contaminated sites as a resource of microorganisms that are expected to advance biotechnological production of PHAs. Employment of such bacteria combines bioremediation with the production of a high value-added material. So far, bacterial strains that belong to the genus of *Sphingobacterium*, *Bacillus*, *Pseudomonas*, and *Rhodococcus* have been isolated and studied regarding their PHA production potential degrading environmental pollutants, as summarized in Table 2 [3,29,33,34,35,36,37,38,39].

*Pseudomonas* species are characterized by their ability to utilize and degrade a variety of carbon sources due to their wide catabolic versatility and genetic diversity. For these reasons, they are a natural choice regarding techniques of in situ and ex situ bioremediation [40]. Several *Pseudomonas* strains have been isolated from hydrocarbon-contaminated soils and together with other *Pseudomonas* sp. have been examined regarding their ability to produce PHA from hydrocarbons. In their study, Nikodinovic et al. [41], investigated PHA accumulation in several *Pseudomonas* strains from single BTEX aromatic substrates and mixed aromatic substrates as well as mixtures of BTEX with styrene. It was reported that when *P. putida* F1 was supplied with 350 μL of toluene, benzene, or ethylbenzene it accumulated PHA up to 22, 14, and 15% of its CDW respectively, while no growth was observed when *p*-xylene and styrene were supplied as the sole carbon source. In the case of *P. putida* mt-2 no growth was obtained with benzene, ethylbenzene, or styrene but when toluene and *p*-xylene were used its PHA content was 22% and 26% respectively. *P. putida* CA-3 efficiently degraded styrene but could not metabolize any of the other hydrocarbons investigated. However, a defined mixed culture of *P. putida* F1, mt-2, and CA-3 was successfully used for PHA production from BTEX and styrene mixtures, where the highest biomass concentration was achieved and PHA content reached up to 24% of the CDW. In another study, strains from petroleum-contaminated soil samples were screened on their ability to degrade toluene and synthesize mcl-PHA [42]. Among them *P. fluva* TY16 was selected to be further investigated. It was shown that the highest PHA content of 68.5% was achieved when decanoic acid was used as the carbon source. In the case of benzene, toluene, and ethylbenzene PHA contents reached up to 19.1, 58.9, and 28.6% respectively, using a continuous feeding strategy. *Pseudomonas* sp. TN301 was isolated from a river sediment sample from a site in a close proximity to a petrochemical industry [43]. Both monoaromatic and polyaromatic hydrocarbons were examined as PHA precursors and cellular mcl-PHA contents varied between 1.2% and 23% of its CDW, while this study was the first one on the ability of a bacterial strain to convert polyaromatic hydrocarbon compounds to mcl-PHA. Moreover, *Pseudomonas* strains isolated from contaminated soil and oily sludge samples from Iranian southwestern refineries accumulated 20–23% of their CDW to mcl-PHA using 2% v/v crude oil as the sole carbon source [32].

As mentioned before, styrene—used for the synthesis of polystyrene—is a major and toxic environmental pollutant. Ward et al. [29], has reported that *P. putida* CA-3 was capable of converting styrene, its metabolic intermediate phenylacetic acid and glucose into mcl-PHA under nitrogen limited conditions, characterized by conversion yields of 0.11, 0.17, and 0.22 g/g, respectively. However, higher cell density and PHA production, characterized by a conversion yield of 0.28 g/g, were observed when cells were supplied with nitrogen at a feeding rate of 1.5 mg/L/h [37]. Moreover, in a recent study the key challenges of improving transfer and increasing supply of styrene, without inhibiting bacterial growth, were addressed [35]. It was shown that by changing the feed from gaseous to liquid styrene, through the air sparger, release of styrene was reduced 50-fold, biomass concentration was five times higher, while PHA production was four-fold compared to previous experiments, with a PHA content reaching up to 32% in terms of CDW and a conversion yield of 0.17 g/g.

A two-step chemo-biotechnological approach has been proposed for the management of post-consumer polystyrene, involving its pyrolysis to styrene oil and subsequent conversion of the styrene oil to PHA by *P. putida CA-3* [44]. According to their results, after 48 h 1.6 g of mcl-PHA were obtained from 16 g of oil with a cellular content of 57%. Following the same approach, the solid fraction of pyrolyzed polyethylene terephthalate (PET) was used as feedstock for PHA production by bacteria isolated from soil exposed to PET granules at a PET processing plant [45]. The isolated strains were identified and designated as *P. putida* GO16, *P. putida* GO19, and *P. frederiksbergensis* GO23 and they were able to accumulate mcl-PHA up to 27, 23, and 24% of their CDW respectively, using 1.1 g/L sodium terephthalate as the sole carbon source under conditions of nitrogen limitation. Recently, conversion of polyethylene (PE) pyrolysis wax to mcl-PHAs was investigated employing *P. aeruginosa* PAO-1 [46]. Addition of rhamnolipid biosurfactants in the growth medium had a positive impact on bacterial growth and PHA accumulation. Substitution of ammonium chloride with ammonium nitrate led to faster growth and earlier PHA accumulation that reached up to 25% of its CDW.

A series of studies has been focused on the degradation of textile dyes for PHA production using *Sphingobacterium*, *Bacillus*, and *Pseudomonas* species. When the dye Direct Blue GLL (DBGLL) was used, *Sphingobacterium* sp. ATM completely decolorized 0.3 g/L in 24 h, while simultaneous polyhydroxyhexadecanoic acid (PHD) occurred reaching up to 64% of its CDW [38]. The potential of *B. odyssey* SUK3 and *P. desmolyticum* NCIM 2112 was also investigated. It was shown that both strains were able to decolorize 0.05 g/L DBGLL by 82% and 86% and produce PHD up to 61 and 52% of their CDW, respectively. In another study, 82% decolorization of 0.8 g/L of the textile dye Orange 3R was feasible, employing *Sphingobacterium* sp. ATM which resulted in the production of 3.48 g/L of PHD and a cellular PHA content of 65% after 48 h [39]. In addition, full decolorization of 0.5 g/L of the textile dye Direct Red 5B (DR5B) was accomplished when the medium was supplemented with glycerol, glucose, starch, molasses, frying oil, and cheese whey. In those cases PHD accumulation was 52, 56, 55, 64, 46, and 10% of its CDW respectively [47].

### 2.3. Halophiles

Halophiles are microorganisms that require salt for their growth and are categorized, according to their halotorerance, in two groups: moderate (up to 20% salt) and extreme (20–30% salt) halophiles [48]. Their name comes from the Greek word for ‘salt-loving’ [49] and can be found in the three domains of life: Archaea, Bacteria, and Eukarya. They thrive in marine and hypersaline environments around the globe such as the saline lakes, salt marshes, and salterns [50,51]. With the use of halophiles the risk for contamination is reduced and/or eliminated since non-halophilic microorganisms cannot grow in media containing high salt concentrations. This is of great importance since their use combines the advantages of low energy requirements under unsterile conditions, minimal fresh water consumption, due to its substitution with seawater for medium preparation, and the possibility of operating contamination free continuous fermentation processes that are much more efficient. In addition, downstream processing cost can be reduced by treating the bacterial cells with salt-deficient water in order to cause hypo-osmotic shock [52]. The above, together with the valorization of low cost substrates, bring halophilic bacteria a step closer to being used as biocatalysts for industrial PHA production.

PHA accumulation by halophilic archaea was first observed in 1972 by Kirk and Ginzburg [53]. So far, the most well-known and best PHA halophilic archaeon producer is *Haloferax mediterranei*, which was first isolated from seawater evaporation ponds near Alicante in Spain [54]. Several studies have shown their ability to accumulate high PHA contents utilizing low cost feedstocks. Among them, vinasse, a byproduct of ethanol production from sugarcane molasses, has been utilized [55]. After pre-treatment, via adsorption on activated carbon, 25% and 50% v/v of pre-treated vinasse led to cellular PHA contents of 70% and 66%, respectively. Maximum PHBV (86% HB–14% HV) concentration reached up to 19.7 g/L, characterized by a volumetric productivity of 0.21 g/L h and a conversion yield of 0.87 g/g, for the case where 25% pre-treated vinasse was used. In another study, stillage derived from a rice-based ethanol industry, was investigated [56]. PHA accumulation was 71% of its CDW that led to 16.4 g/L of PHBV (85% HB-15% HV) with a yield coefficient of 0.35 g/g and a volumetric productivity of 0.17 g/L h. Moreover, cheese whey hydrolysate—obtained upon acid pre-treatment—has been used for the production of PHBV with low HV fraction, 1.5 mol % [57]. Batch cultivation of *H. mediterranei* led to the production of 7.54 g/L of biomass, with a PHA content of 53%, and a volumetric productivity of 0.17 g/L h. Olive mill wastewater (OMW), which is a highly polluting waste, was also utilized recently as the sole carbon source for PHA production [58]. Using a medium containing 15% of OMW up to 43% of PHBV/CDW was produced consisting of 6 mol % HV in a one-stage cultivation step.

Halophilic bacteria belong to γ-Proteobacteria and they can grow on a wide range of pH, temperature, and salinity concentrations up to 30% (w/v NaCl) and possess the ability to accumulate PHA [52]. *Halomonas* TD01 has been isolated from Aydingkol Lake in China. This strain was investigated regarding its PHA production potential under unsterile and continuous conditions [59]. Glucose salt medium was used and initial fed-batch cultivation resulted in the production of 80 g/L of biomass with a PHA content of 80%, in the form of PHB, after 56 h. A continuous and unsterile cultivation process was developed that lasted for 14 days, and that allowed cells to grow to an average of 40 g/L containing 60% PHB in the first reactor. Cells were forwarded by continuous pumping from the first to the second reactor that contained nitrogen-deficient glucose salt medium. In the second reactor PHB levels ranged from 65 to 70% of its CDW and a conversion yield of 0.5 g/g was achieved. This was the first attempt for continuous PHA production under non-sterile conditions from a halophilic bacterium. In addition, Yue et al. [60] explored the potential of *Halomonas campaniensis* LS21, isolated from the Dabancheng salt lake in China, to produce PHA in a seawater-based open and continuous process. The strain utilized a mixture of substrates mainly consisting of cellulose, starch, animal fats and proteins. Instead of fresh water fermentation was performed using artificial seawater composed of 26.7 g/L NaCl, among others under a pH around 10 and 37 °C. PHB accumulation reached up to 26% during 65 days of continuous fermentation without any contamination. Through this study the benefits of long-lasting, seawater-based, and continuous processes for PHA production under unsterile conditions were demonstrated.

*Bacillus megaterium* has recently drawn attention since several studies have isolated such stains from salterns. *Bacillus megaterium* H16, isolated from the solar salters of Ribandar Goa in India, was shown to accumulate up to 39% PHA in the presence (5% w/v) or absence of NaCl using glucose [61]. In another study, a mangrove isolate that was found to belong to *Bacillus* sp. could tolerate salinity up to 9% w/v [62]. The certain strain was able to utilize a wide variety of carbon sources such as monosaccharides, organic acids, acid pre-treated liquor, and lignocellulosic biomass reaching cellular PHA contents of up to 73% of its CDW. Furthermore, *Bacillus megaterium uyuni* S29, isolated from Uyuni salt lake in Bolivia, was examined in terms of its salinity tolerance and impact on biomass and PHB production [63]. It was observed that the strain could grow at 10% w/v NaCl while PHB production was observed even at high salinity levels of 25% w/v. Optimum results for biomass and PHB production were achieved in medium containing 4.5% w/v NaCl and were 5.4 and 2.2 g/L characterized by a yield coefficient of 0.13 g/g and a volumetric PHB productivity of 0.10 g/L h.

The results obtained from the studies described above are very promising and demonstrate the remarkable potential of halophiles for biotechnological production of PHAs. Although, processes performed under high salinity concentrations present disadvantages such as the corrosion of stainless steel fermenters and piping systems [51,52]. However, since no sterilization is required when halophiles are used, other types of low cost materials, such as plastics and ceramics, may be used to design and construct fermentation and piping systems in order to overcome corrosion issues. In addition, the number of halophilic bacteria that their genome is being sequenced is constantly increasing throughout the years. Subsequently, in the near future, molecular biology techniques will result in metabolically engineered strains with better performances regarding their industrial application [48,51].

### 2.4. Synthetic Biology of PHA Producers

Synthetic biology tools may aid in developing competitive bioprocesses by engineering biocatalysts with the potential of being employed at industrial scale, producing large amounts of PHA at low prices [64]. Industrial biotechnology requires non-pathogenic, fast growing bacteria that do not produce toxins and their genome is easily manipulated. Utilization of cellulose and fast growth under a wider range of temperature and pH are considered a plus [65]. The effort of minimizing PHA production cost focuses mainly on engineering strains that show higher PHA production efficiency from raw waste material, require less energy consumption during PHA production, simplify downstream processing, and produce tailored functional polymers for high value-added applications.

In order to achieve high PHA volumetric productivities high cell densities need to be obtained of up to 200 g/L, characterized by high cellular PHA contents, above 90% g PHA/g CDW. Manipulation of genes related to the oxygen uptake, quorum sensing, and PHA biosynthetic mechanisms may enhance PHA production [65]. For example, oxygen limitation may occur, after obtaining high cell densities, in order to initiate/promote PHB production. In a relevant study, anaerobic metabolic pathways were designed in *E. coli* (over-expressing hydrogenase 3 and acetyl-CoA synthatase) to facilitate production of both hydrogen and PHB. In that way, the formation of toxic compounds such as formate and acetate was avoided by driving carbon fluxes towards the production of PHB. The engineered strain showed improved hydrogen and PHB production. In addition, PHB pathway optimization has been also investigated in *E. coli* by adjusting expression levels of the three genes *phbC*, *phbA*, and *phbB* [66]. *phbCAB* operon was cloned from the native PHA producing strain *Ralstonia eutropha*. Rational designed Ribosomal Binding Sites (RBS) libraries were constructed based on high or low copy number plasmids in a one-pot reaction by an Oligo-Linker Mediated Assembly method (OLMA). Bacterial strains accumulating cellular contents of 0 to 92% g PHB/g CDW were designed and a variety of molecular weights ranging between 2.7–6.8 × 10^6^ was achieved. The certain study demonstrated that this semirational approach combining library design, construction, and proper screening is an efficient tool in order to optimize PHB production.

Another example where synthetic biology has been implemented are halophilic bacteria, which allow for PHA production under continuous mode and unsterile conditions. These features increase the competitiveness of industrial PHA production. In addition, halophilic bacteria have been proven easy for genetic manipulation, thus allowing for the construction of a hyper-producing strain [65,67]. For example, both recombinant and wild type *Halomonas campaniensis* LS21were able to grow on mixed substrates (kitchen wastes) in the presence of 26.7 g/L NaCl, at pH 10 and temperature of 37 °C continuously, for 65 days, without any contamination. Recombinant *H. campaniensis* produced almost 70% PHB compared to wild type strain that in which PHA accounted for 26% of its CDW [60].

Engineering the morphology of bacteria, in terms of cell size increase, has been recently investigated. Apart from PHA granules, several bacteria may accumulate polyphosphates, glycogen, sulfur, or proteins within their cells that limit cell space availability. In order to increase cell size, approaches such as deletion or weak expression of on actin-like protein gene mreB in recombinant *E. coli* resulted in increasing PHB accumulation by 100% [68,69,70]. In addition, manipulating PHA granule-associated proteins leads to an increase in PHA granule size allowing for easier separation [71].

Intracellular accumulation of PHA necessitates several extraction and purification steps. Synthetic biology approaches have been developed to control and facilitate the release of PHA granules to the medium. For example, the programmed self-disruptive strain *P. putida* BXHL has been constructed in a recent study, deriving from the prototype *P. putida* KT2440 which is a well-known mcl-PHA producer [72]. This was based on a controlled autolysis system utilizing endolysin Ejl and holing Ejh isolated from EJ-1 phage and in order to improve the efficiency of the lytic system this was tested in *P. putida* tol-pal mutant strains with alterations in outer membrane integrity. According to results, it was shown that the engineered lytic system of *P. putida* BXHL provided a novel approach to inducing controlled cell lysis under PHA producing conditions, either produce PHA accumulating cells that were more susceptible to lytic treatments. The certain study demonstrated a new perspective on engineered cells facilitating PHA extraction in a more environmentally friendly and economic way.

PHA structures include PHA homopolymers, random and block copolymers, and also different monomer molar fractions in copolymers. Block copolymers have been reported regarding their resistance against polymer aging. This is of crucial importance since slower degradation of polymer occurs leading to better performance and consistent polymer properties [73]. It has been observed that downregulating of *β*-oxidation cycle in *P. putida* and *P. entomophila* may be used for controlling PHA structure when fatty acids are used as precursors for PHA production [73,74,75,76]. In the case of fatty acids, containing functional groups are consumed by bacteria, introduction of those functional groups into PHA polymer chains occurs [77]. In addition, recombinant strains of *E. coli* have been constructed for the synthesis of block polymers with superior properties [78,79,80,81]. PHA diversity is possible by engineering basic biosynthesis pathways (acetoacetyl-CoA pathway, in situ fatty acid synthesis, and/or *β*-oxidation cycles) as well as through the specificity of PHA synthase [82].

## 3. PHA Production by Mixed Microbial Consortia (MMC)

Currently, industrial PHA production is conducted using natural isolates or engineered strains and pure substrates [83,84]. An alternative scenario that would contribute to the reduction of the PHA production cost is to employ mixed culture biotechnology [85,86]. This approach uses open (under non-sterile conditions) mixed microbial consortia (MMC) and ecological selection principles, where microorganisms able to accumulate PHA are selected by the operational conditions imposed on the biological system. Thus, the principle is to engineer the ecosystem, rather than the strains, combining the methodology of environmental biotechnology with the goals of industrial biotechnology [87]. The cost reduction derives mainly from operations being performed under non-sterile conditions, and their consequent energy savings, and the higher adaptability of MMC to utilize waste streams as substrates.

**Figure 1:**
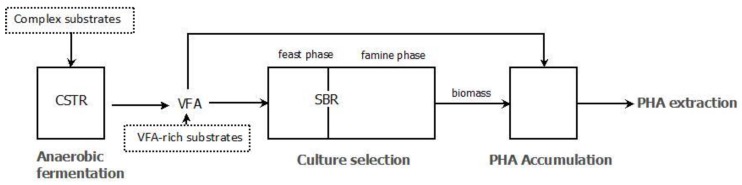
PHA production process by mixed microbial cultures. Modified from [88]. CSTR: continuous stirred tank reactor, SBR: Sequential Batch Reactor.

Processes for PHA production in mixed cultures are usually performed in two steps (Figure 1). In the first step, SBR reactors (sequential batch reactors) are used to select and enrich a microbial population with high PHA production capacity by applying transient conditions. In the second step, the culture from the SBR is subjected to conditions maximizing the PHA accumulation, from where cells are harvested for PHA extraction and purification when they reach maximum PHA content [88,89].

Unlike pure cultures, where glucose is mostly used as a substrate for PHA production, mixed culture biotechnology makes use of volatile fatty acids (VFA) as the precursors for PHA production [90]. The main reason is that carbohydrates in MMCs, as well as other substrates such as glycerol, tend to form glycogen besides PHA [91,92]. For those substrates, a previous step is generally included (Figure 1), during which they are fermented into volatile fatty acids (VFA) in continuous mode (CSTR). Moreover, this is also applied to complex substrates, such as olive mill wastewater [10,93,94], cheese whey [95], and other food wastes [96] in order to obtain a more homogeneous readily available feed for the PHA production. VFA conversion into PHA require few steps, and usually presents very high yields and rapid uptake rates [88,97]. This is especially the case for butyric acid, which has now been reported in many studies as the VFA presenting the highest yields (up to 0.94 Cmol PHA/Cmol S) and being the one preferably up-taken by MMCs [2,98,99,100]. As a matter of fact, butyric acid preference has been observed even in cultures that were not exposed to it during the enrichment [93].

It is worth mentioning that the distribution of VFA is known to affect the PHA monomer composition, where VFA with an even number of carbon atoms tend to produce PHB while VFA with odd carbon atoms tend to produce PHBV copolymers with different % HV molar fractions [101]. Based on this fact, many studies have suggested the possibility of regulating the PHA composition by manipulating the fermentation conditions in the preceding acidogenesis step [2,95,100].

This section provides an overview of the different types of existing enrichment techniques, performed within the last 10 years. Each one of the enrichment strategies presents different advantages; either related to the cost of the process or to increased cellular PHA content. Thus, they all present opportunities to further improve economic and sustainable PHA production. Such opportunities are commented for each of the enrichment techniques. This section is followed by a compilation of recent advances regarding the PHA accumulation stage, aiming at increasing the productivity. Finally, recent attempts to bring MMC to pilot scale are described, followed by a section highlighting the main challenges and bottlenecks of the MMC.

### 3.1. Types of Enrichments

Until the late 2000s, two types of enrichments dominated the research panorama related to PHA mixed culture biotechnological production, namely the anaerobic/aerobic selection and aerobic dynamic feeding. These types of enrichments have already been previously reviewed in other articles [88,102,103,104], thus apart from a general description of the mechanisms, only the recent trends are described in the respective Section 3.1.1 and Section 3.1.2. The following sections are dedicated to recent (and still less widespread) types of enrichments developed within the last decade. The main characteristics of all types of enrichments described are summarized in Table 3.

#### 3.1.1. Anaerobic/Aerobic Enrichments (AN/AE)

PHA production from MMC was first observed in biological phosphate removal by activated sludge in wastewater treatment plants, where aerobic and anaerobic steps alternate [90]. Thus, the first attempts of enriching a PHA storing community were performed by replicating those conditions. In these cases, Polyphosphate Accumulating Organisms (PAOs) and Glycogen Accumulating Organisms (GAOs) were described to accumulate PHA during the anaerobic phase, where the electron acceptor becomes limiting. In the aerobic phase, these microbes would consume the internally stored PHA, using the available oxygen, obtaining thus a higher adenosine triphosphate (ATP) yield compared to the substrate being metabolized anaerobically [102,104]. Nevertheless, as substrate catabolism and PHA formation, during the anaerobic phase, requires ATP and reducing equivalents, these microbes would also depend on the accumulation of glycogen or polyphosphate from the stored PHA during respiration, that limit the PHA production capacity of the cultures. Maximum PHA contents of around 20% had been reported from these consortia enriched under AN/AE conditions. However, in the late 1990s the aerobic dynamic feeding (ADF) enrichment was introduced, obtaining higher PHA contents [102,103,104], and thus, research efforts in AN/AE remained limited. Nevertheless, recent developments in PHA production following AN/AE enrichment, demonstrated that PHA might be also accumulated aerobically, without depending on glycogen and phosphate reserves, and high PHA storage capacities (up to 60%) could be obtained [105]. Thus, further research could prove the feasibility of AN/AE that would demonstrate benefits of saving energy costs, in terms of aeration requirements, compared to ADF.

#### 3.1.2. Aerobic Dynamic Feeding (ADF)

In enrichment under ADF conditions the limiting factor, promoting PHA accumulation, is carbon substrate availability rather than the electron acceptor [104]. This process relies on subsequent feast/famine cycles where the culture is subjected initially to an excess of carbon source, and then submitted to carbon deficiency under aerobic conditions. Bacteria that are able to convert carbon to PHA during the feast phase have a competitive advantage towards the rest of the microbial population, as they utilize PHA as a carbon and energy reserve during the famine phase, allowing them to grow over non-PHA storing microorganisms [104,105]. Moreover, a limitation of internal factors, such as RNA and enzymes required for growth, seems to be crucial [102]. In order for cells to grow, a considerable amount of RNA and enzymes are needed, which might not be available after long starvation periods. Nevertheless PHA synthase, the key enzyme for PHA polymerization, is active during PHA production and degradation, generating a futile cycle that wastes ATP but enables the PHA mechanism to be ready when a sudden addition of carbon occurs, providing them with higher responsiveness [106,107,108]. In this way, a new competitive advantage of PHA producers arises, given that they can use PHA to regulate substrate consumption and growth [106]. PHA contents up to 90% of the dry cell weight have been reported using this strategy [89,109], higher than the ones reached following AN/AE enrichment [102,103,104].

Most of the studies performed within the last 10 years have been based on ADF enrichment. Apart from investigating the feasibility of this strategy using a variety of substrates, recently reviewed by Valentino and colleagues [97], the main focus has been on evaluating the impact of different parameters during the enrichment process. An overview on how those parameters influence PHA production from MMCs has been recently reported on, including the effect of hydraulic retention time (HRT), solids retention time (SRT), pH, temperature, nitrogen concentration, dissolved oxygen concentration (DO), cycle length, influent concentration, feast/famine ratio, and food/microbe ratio [110]. Regarding process configuration, a continuous system has been proposed where instead of an SBR, the feast and the famine phases were operated in separate CSTR [111]. Although no significant improvements were observed with respect to the conventional SBR configuration, the study demonstrated that successful enrichment was also possible in continuous mode, which is considered advantageous in the case of putative coupling to the following PHA accumulation step under continuous mode.

#### 3.1.3. Variations of the ADF Enrichments

Even though microbial communities with high PHA-storing capacity have been obtained with several parameter combinations, the presence of non-PHA accumulating microorganisms is still not completely avoided. This is partially due to the presence of organic content other than VFA present in waste streams, allowing the growth of non-PHA accumulating bacteria [112]. A possible way to overcome the presence of such bacteria has been recently proposed where the culture was settled and the supernatant was discharged just after VFA depletion. In this way, consumption of the remaining organic matter—measured as chemical oxygen demand (COD)—and growth of side-population was prevented while the fraction of PHA-producers was considerably increased as verified by molecular techniques [113]. An increase in the PHA content from 48% to 70% was observed by applying this strategy. The authors suggested that, apart from the role of the remaining COD, also the increased cell density of PHA packed cells enhanced the enrichment, as those cells would have a higher tendency to settle after the feast phase. This observation coincided with the results obtained using only acetate as a carbon source, where the settling after the feast phase also increased the PHA accumulation capacity of the culture from 41% to 64–74% [114]. As no residual COD was present in those experiments, the effect could be entirely attributed to differences in the cell density that led to an additional physical selection. 

Similarly, the growth of such non-PHA accumulating bacteria was observed to be restricted by applying nitrogen limitation [112]. Following this reasoning, a variation of the ADF was recently proposed, where also a nitrogen deficiency was imposed during the feast phase, while providing nitrogen during the famine phase to enable growth from the accumulated PHA. Thus, an uncoupling of the carbon and nitrogen took place in the SBR [2,86,115]. This strategy resulted in higher PHA contents at the end of the feast phase using synthetic VFA [2,115], cheese whey [86], and 1,3-propanediol from fermented crude glycerol [116] as a substrates. When a separate PHA accumulation was performed with the cultures of such enrichments, higher PHA yields and productivities were obtained with this strategy compared to carbon-nitrogen coupled ADF [86]. This strategy also allowed for a more stable system for long term operation. Nevertheless, given that the enrichment is already performed at conditions maximizing PHA accumulation, it was also suggested that a separate PHA accumulation might no longer be required if part of the biomass is already harvested after the feast phase, something that would significantly contribute towards the reduction of the costs of the process [115]. Moreover, given that there is already a selective pressure in the feast phase, the duration of the famine phase would be less important, so as to achieve an effective selection, and this could enable a reduction of its duration leading to enhanced process productivity [86].

#### 3.1.4. ADF Enrichments in Halophilic Conditions

As previously discussed, the use of halophilic bacteria comes with various advantages. PHA production using halophilic bacterial populations can be performed using seawater instead of fresh or distilled water or using high salinity wastewater produced by several industries, namely food processing industries. In addition, halophilic bacteria can be lysed in distilled water thus reducing downstream processing costs due to lower quantities of solvents required. Enrichment of a halophilic PHA accumulating consortium under ADF conditions has been recently investigated using different carbon sources as substrate, which resulted in cellular PHA contents reaching up to 65% and 61% PHA using acetate and glucose respectively, demonstrating the potential of this strategy in MMC [117]. Recently, a previously enriched MMC fed with a mixture of VFAs containing 0.8 g/L Na^+^ was examined regarding its PHA accumulation capacity under transient concentrations of 7, 13, and 20 g/L NaCl [118]. Since the particular MMC was not adapted to saline conditions, PHA accumulation capacities and rates decreased with higher NaCl concentrations while biopolymer composition was affected in terms of HB:HV ratio.

#### 3.1.5. Mixed Photosynthetic Consortia

A new approach relying on the photosynthetic activity of mixed consortia has been explored recently [119,120,121,122]. Based on the previous observation of PHA production in photosynthetic strains, an illuminated SBR operating without aeration was proposed, eliminating the costly need for aeration during ADF enrichments. In such a system, photosynthetic bacteria uptake an external carbon source, in the form of acetate, during the feast phase using light as an energy source. PHB was produced at the same time as a sink of NADH, given that no electron acceptor was present. During the famine phase PHB was consumed using oxygen as an electron acceptor, which was not provided though aeration but produced by algae also present in the SBR. Under these conditions, up to 20% PHB was attained during the PHA accumulation step [119], which was also possible by utilizing other VFAs such as propionate and butyrate [121]. However, it was observed that a dark feast phase could also be envisioned, given that similarly to AN/AE enrichments, glycogen accumulation occurred during the famine phase, which was subsequently used as a complementary energy source to uptake acetate. With this SBR configuration, which would considerably reduce the need of illumination, a 30% PHA was accomplished [122].

The best PHA productivity though, was obtained in a system operating in a permanent illuminated feast phase (instead of successive feast-famine cycles) without oxygen supply [120]. This was based on the fact that photosynthetic accumulating bacteria out-compete other bacteria and algae without the need of transient presence of carbon source. Therefore, productivity was significantly increased due to the elimination of the famine phase and since there was no need for a separate PHA accumulation reactor. However, considerable input of light was required in order to obtain cultures with high PHA content (up to 60% of the dry weight), so the economic advantages of such systems should be further explored. Nevertheless, this process will allow significant savings in energy, since no sterilization and aeration are required, which will have an impact on the final price of the polymer.

#### 3.1.6. Aerobic–Anoxic Enrichment Coupled with Nitrification/Denitrification

Basset and colleagues [123] developed a novel scheme for the treatment of municipal wastewater integrating nitrification/denitrification with the selection of PHA storing biomass, under an aerobic/anoxic and feast/famine regime. The process took place in a SBR (where NH_4_^+^ is converted into NO_3_^−^ with a simultaneous selection of PHA storing biomass—and with COD being converted to PHA) and the subsequent PHA accumulation in a batch reactor (where PHA is consumed to allow denitrification, under famine–anoxic conditions, without the need of external addition of organic matter). The carbon source added during the selection and accumulation steps consisted of fermentation liquid from the Organic Fraction of Municipal Solids Waste (OFMSW) and primary sludge fermentation liquor.

The advantage of this approach is that the potential for recovering biopolymers from wastewater presents particular interest, when the latter is integrated within the normal operation of the plant. An important benefit of this strategy is that anoxic denitrification usually requires a carbon source, which at that point is usually low and already consumed in a wastewater treatment plant (WWTP). In this case however, it can occur without external addition of carbon source by using those stored internally in the form of PHA. 

Results showed that during SBR operation ammonium oxidation to nitrite reached on average 93.4 ± 5.25%. The overall nitrogen removal was 98% (resulting in an effluent with only 0.8 mg NH_4_^−^ N L^−1^). Similar results were obtained by Morgan-Sagastume et al. [124]. Denitrification efficiency and rate did not seem to be affected by the carbon source. When sufficient PHA amount was available, denitrification of all available nitrate was observed. COD removal reached up to 70% when DO level was higher (2–3 mg L^−1^). PHA content decreased during nitrification due to the lack of external COD. However, after complete nitrification, there was enough PHA to carry out the denitrification process. Even though biomass was rich in PHA storing bacteria, PHA accumulation reached only 6.2% during the feast phase (first 10 min) and it was progressively consumed before the initiation of the anoxic phase to 2.3%, which was enough to complete the subsequent denitrification. Nevertheless, the selection of PHA storing biomass under feast (aerobic)–famine (anoxic) conditions required less DO compared to the typical feast–famine regime carried out under continuous aerobic conditions, leading to a reduction of 40% of the energy demand.

The PHA accumulation capacity of the biomass, previously selected in the SBR, was further evaluated in accumulation batch reactors with the use of OFMSW, and primary sludge fermentation liquid. After 8 h of accumulation with OFMSW, the stored PHA was 10.6% (wt.). In the case where fermented sludge liquid and OFMSW was used as carbon source, the contribution to growth was higher, due to the elevated nutrient content, and the lower VFA/COD ratio but only 8.6% PHA was accumulated after 8 h. The carbon source was proven to play an important role in the PHA accumulation step as the presence of non-VFA COD contributed to the growth of non-PHA-storing biomass [124,125]. PHA storage yields could be potentially improved with a more efficient solid–liquid separation after the fermentation process.

#### 3.1.7. Anoxic–Aerobic Strategy Coupled with Nitrification/Denitrification

Anoxic–aerobic enrichments coupled with denitrification, where nitrate is used as electron acceptor during PHA accumulation, has been already explored using synthetic VFA since early 2000s [103]. A recent study applying this strategy has been reported which investigated the use of the condensate and wash water from a sugar factory [126]. Furthermore, they considered that, in combination with harvesting enriched biomass from the process water treatment, side-streams could be exploited as a substrate for PHA accumulation. This approach (together with the aerobic–anoxic strategy shown in Section 3.1.6) would have the big advantage of significantly reducing the PHA production costs, through the integration of already existing full-scale WWTP and reduction of aeration needs.

In that study [126], they used parallel SBRs fed alternatively with condensate and wash water, developing a microbial consortium that removes inorganic nitrogen by aerobic and anoxic bioprocess steps of nitrification and denitrification. Alternating bioprocess conditions of anoxic feast (supporting denitrification) and aerobic famine (supporting nitrification) in mixed open cultures was expected to furnish a biomass with stable PHA accumulating potential characterized by its ability to remove carbon, nitrogen, and phosphorus in biological processes. In more detail, one laboratory SBR was operated with suspended activated sludge (AS) and long SRT, similar to the full-scale (SRT > 6 days), while the other SBR was a hybrid suspended activated sludge and moving bed biofilm reactor (MBBR) with short SRT of 4–6 days. MBBR technology employs thousands of polyethylene biofilm carriers operating in mixed motion within an aerated wastewater treatment basin. Therefore, the MBBR-SBR was used as a means for maintaining nitrifying activity while enabling enrichment of biomass at relatively low SRT.

The results showed a COD removal performance of 94% and 96% for AS- and MBBR-SBRs, respectively. Full nitrification was achieved in both systems, with exception of periods showing phosphorous or mineral trace element limitation. Soluble nitrogen removal reached 80 ± 21% and 83 ± 11% for AS- and MBBR-SBRs, respectively. MBBR-SBR showed more stable performance under lab-scale operation. The process achieved a PHA content of 60% g PHA/g VSS in both cases. A significant advantage was the possibility of lowering the SRT while maintaining a robust nitrification activity and improving the removal of soluble phosphorus from the process water.

#### 3.1.8. Microaerophilic Conditions

In 1998, Satoh and colleagues [127] investigated the feasibility of activated sludge (from laboratory scale anaerobic–aerobic reactors) for the production of PHA, by optimizing the DO concentration provided to the system. They were able to obtain a PHA content of around 20% in anaerobic conditions and 33% under aerobic conditions. When applying a microaerophilic–aerobic process, by supplying a limited amount of oxygen into the anaerobic zone, they were able to increase the PHA accumulation to 62% of sludge dry weight.

PHA production using palm oil mill effluent (POME) was investigated by Din and colleagues [128], using a laboratory SBR system under aerobic feeding conditions. The microorganisms were grown in serial configuration under non-limiting conditions for biomass growth, whereas in the parallel configuration the nutrient presence was controlled so as to minimize biomass growth in favor of intracellular PHA production. PHA production under aerobic, anoxic, and microaerophilic conditions was investigated and it was shown that PHA concentration and content increased rapidly at the early stages of oxygen limitation while the production rate was reduced at a later stage implying that oxygen limitation would be more advantageous in the PHA accumulation step.

Another interesting study was published by Pratt and colleagues [129], where the effect of microaerophilic conditions was evaluated during the accumulation phase, using an enriched PHA culture, harvested from a SBR fed with fermented dairy waste. Batch experiments were conducted to examine the effect of DO on PHA storage and biomass growth. The results showed that in microaerophilic conditions a higher fraction of substrate was accumulated as PHA, compared to high DO conditions. Also, the intracellular PHA content was 50% higher during early accumulation phase. Interestingly, the accumulation capacity was not affected by the DO, despite its influence on biomass growth. The PHA content in both low and high DO concentrations reached approximately 35%. However, the time needed to achieve maximum PHA content at low DO level was three times longer than in the case of high DO concentration. The reason why PHA accumulation was proven to be less sensitive to DO, compared to its effect on biomass growth, was explained by the fact that low DO levels limit the availability of ATP, while high DO supply provides surplus ATP and high growth rates (and consequently reduced PHA yield). In addition, when MMCs were fed with multiple VFAs (acetate, propionate, butyrate, and valerate) it was also shown that, during PHA accumulation, high DO concentration is required to reach maximum PHA accumulation rates due to low specific VFA uptake rates under low DO levels [130].

The effect of dual nitrogen and DO limitation has been also investigated in MMCs fed with a VFA mixture of acetate, propionate, and butyrate and acidified OMW [10,99]. As discussed above, it was shown that during the PHA accumulation step, under batch mode, lower substrate uptake and PHA production rates were obtained compared to assays performed under nitrogen limitation. Moreover, PHA accumulation percentages and the yield coefficient Y_PHA/S_ was lower in the case of dual limitation, while the accumulation of non-PHA polymers within the cells was indicated.

Those reports demonstrate that manipulating oxygen concentration could influence growth and PHA storage. Manipulating DO instead of limiting nitrogen or phosphate availability could represent a significant opportunity for PHA production processes that utilize nutrient rich feedstocks. A major advantage of operating at low DO is the reduced aeration requirements leading to reduction of operating costs. However, this advantage can be countered by the fact that PHA accumulation in low DO environments can be significantly slower.

#### 3.1.9. PHA Accumulation without Previous Enrichment

The three-step process described in Figure 1, consisting in an enrichment step followed by an accumulation step, has been proven efficient to obtain cultures with high PHA contents. Nevertheless, several authors have put in doubt the alternative of having a separate enrichment step. The main reason is that during the enrichment, PHA is produced, but it is also allowed to be consumed to drive the selection. Thus, this step consumes substrate without leading to any net production of PHA, lowering the overall PHA/Substrate yields of the system [97,131]. Thus, skipping this step could imply considerable improvements on those parameters.

Already in 2002, PHA accumulation without a previous enrichment step using activated sludge was reported to obtain PHA contents up to 30% [132]. Later on, fed-batch cultivation under nitrogen limiting conditions was reported to obtain up to 57% PHA [133]. Cavaillé and colleagues performed fed-batch PHA accumulation experiment as well using activated sludge without previous enrichment, but applying phosphorous limitation and achieved up to 70% PHA [134]. Substrate to PHA yield reached up to 0.2 Cmol PHA/Cmol S using acetic acid. This yield was considerably lower than that obtained in PHA accumulation steps submitted to a previous enrichment step (up to 0.9 Cmol PHA/Cmol S), but comparable to the overall yields of enrichment and accumulation strategies summed up [134]. Moreover, they further developed the system into a continuous process, and attained a stable operation where the cells in the effluent contained 74% PHA [135]. Their findings also evidenced that the continuous system was not stable at severely phosphorous limited experiments, because the growth rate could not be maintained and the cells were washed out of the reactor. The key was that differently from when a separate enrichment is performed, the continuous PHA accumulation without previous enrichment relied on the occurrence of both growth and storage responses. Moreover, the authors suggested that phosphorous limitation might offer more flexibility than nitrogen limitation when both PHA formation and growth are a goal, given that phosphorous is less needed than nitrogen for growth-related metabolism [135].

### 3.2. PHA Accumulation

As in the enrichment step, several operational parameters such the temperature [136] and the pH have an impact on PHA accumulation [2,137,138,139]. Nonetheless, the most critical aspect during PHA accumulation experiments is the cultivation strategy employed. High substrate concentrations supplied under batch mode should be avoided since they can cause inhibition and thus limit PHA productivity [105,140]. In order to circumvent that, several fed-batch strategies have been suggested. Pulsed fed-batch cultivation has been suggested when synthetic VFA mixtures are used [140,141]. However, due to an increase occurring in the working volume after the addition of substrate the feed should be very concentrated. Nevertheless, this is rarely the case with fermentation effluents, since they usually do not exceed 20 g COD/L [97]. Discharge of the exhaust supernatant has been suggested as an alternative [137,142,143] yet, this approach requires a settling step between batches, which severely limits the productivity [137].

Continuous feeding processes have shown the best results until now, given that they can attain a sustained productivity [89,137,143,144]. Pulsed fed-batch production may result in high PHA productivity but as the substrate is being consumed, PHA productivity eventually decreases. This phenomenon might be avoided by supplying substrate in a continuous manner. Continuous substrate addition has been successfully performed using the pH as an indicator, given the pH increases with VFA consumption [89,137,143,144]. On the other hand, less successful results have been obtained when the substrate was supplied, taking into account previously observed substrate uptake rates resulting in either accumulation or limitation of substrate in the reactor [140,141]. Alternatively, an on-demand continuous addition of substrate, based on change of DO, has been proven efficient to maintain optimal amounts of carbon substrate in the reactor [145].

High productivities up to 1.2 g PHA/L h combined with high PHA yields, 0.8 Cmol PHA/Cmol S, have been achieved with continuous-feeding systems [137]. However, similarly to the pulsed fed-batch, such values have been reported only when synthetic substrates of high concentration were present in the feed. Much lower values are reported in real substrates due to the diluted nature of these substrates and the consequent increment in reactor volumes [97]. A way to overcome this could be the development of a continuous feeding scheme for PHA accumulation under low biomass loading rates (3.5–5.5 Cmol VFA/Cmol X/d). So far, this venture has only been investigated once using MMC [139]. The authors suggested a PHA accumulation reactor operating under continuous mode, were the effluent was allowed to settle and the resulting sludge recycled back to the reaction vessel. The system worked with a rather diluted effluent in the feed (around 100 Cmol VFA) and was shown to obtain higher specific productivities than the pulse-fed-batch. Nevertheless, overall productivity of the system was not reported in that study. It is worth noting that the system was not coupled to an SBR operating in continuous mode, so the operation of the reactor was also for a limited period of time.

Regarding the nutrient availability, nitrogen limitation or deficiency is usually reported to improve the PHA yield and content [2,105,146,147]. On the other hand, several studies have concluded that the role of nitrogen during the accumulation step is secondary since PHA storage was preferred over growth regardless of the nitrogen concentration [144,148]. Moreover, in another study it was observed that nitrogen limitation did not enhance the PHA accumulation [149]. As a matter of fact, the main reason for limiting the amount nitrogen is to prevent bacterial growth of non PHA-accumulating bacteria [112]. Hence, different observations from different cultures do not imply contradictions but highlight the fact that the requirement for nitrogen limitation, in order to obtain high PHA contents, is highly dependent on the composition of the enriched culture and the type of substrate fed. In terms of productivity though, nutrient limitation rather than deficiency was reported to show higher productivities [145]. According to a certain study, the absence of an essential nutrient for growth leads to cellular PHA saturation, while nutrient limitation allows cells to duplicate prolonging PHA accumulation without enabling excessive growth response. In addition, it was shown that the best productivities were obtained from dual limitation of nitrogen and phosphorous.

### 3.3. Pilot Scale Experiences

Several industrial/agro-industrial effluents and residues have been investigated so far as potential feedstocks for PHA production. Effluents, rich in sugars, glycerol, and/or fatty acids were either directly used for the selection of PHA accumulating MMCs or were previously fermented for the conversion of carbohydrates to VFAs, the preferable precursors for PHA production using MMCs. Numerous studies on PHA production from industrial effluents have been performed, that have been recently reviewed by Valentino et al. [97]. On the other hand, studies on PHA production in pilot-scale by MMCs are rather scarce and relevant efforts have recently started, in 2010s, while no full-scale production of PHA by MMC exists yet.

A common feature in all pilot-scale studies is that effluents/feedstocks were always fermented prior to PHA production. Also, most efforts on PHA production in pilot-scale focus on integrating and combining PHA production with existing processes in wastewater treatment plants so as to reduce the production cost by exploiting the available infrastructure as much as possible. In this context, anoxic/aerobic MMC selection regimes can be coupled to nitrification and denitrification activities despite the fact that the highest PHA yields and cell content in PHA are usually reported for aerobic dynamic feeding selection regimes. Another tendency in pilot-scale studies is the use of a different effluent for the MMC enrichment than the one fed during the PHA accumulation step. The first pilot-scale study, concerning MMC PHA production, was performed from pre-fermented milk and ice-cream processing wastewater, as reported by Chakravarty et al. [150], with a PHA content of 43% and a PHA yield of 0.25 kg PHA/kg COD being obtained. In 2014, Jia et al. [125] studied the production of PHA in pilot-scale with pre-hydrolyzed and fermented raw excess sludge. In both studies, activated sludge was used as the raw material for its enrichment to PHA accumulating microorganisms. A series of pilot-scale studies was conducted and published with the participation of Anoxkaldnes and Veolia Water Technologies [126,151,152]. In the study of Morgan-Sagastume et al. [151], the potential of waste sludge, generated in wastewater treatment plants as a feedstock for PHA production was evaluated. This was done in the context of integrating PHA production in existent WWTP valorizing at the same time the excess sludge that in general represents a burden for further treatment and disposal. A very interesting point in the study of Bengtsson et al. [152] is that denitrifying microbial biomass was also selected towards high PHA producing potential by applying an anoxic-feast and aerobic-famine selection pattern and therefore the process comprised nitrification and denitrification steps followed by accumulation of PHA. Tamis et al. [153] investigated PHA production from fermented wastewater deriving from a candy bar factory. Activated sludge was enriched in PHA accumulating microorganisms under aerobic feast and famine regime and the obtained PHA content was the highest reported so far in pilot-scale at 70–76%. Table 4 summarizes the main features of the pilot-scale studies published so far. Overall, as it regards pilot-scale studies, their performance cannot really be compared to respective lab-scale studies but they provide valuable information on PHA formation under variable feedstock characteristics and allow production of significant amounts of polymer that can be processed for a full characterization. The variation of feedstock composition combined with the oxygen mass transfer limitations occurring at a larger scale, could be the main reason why PHA yields and cell content in pilot-scale studies are in general lower than the ones reported in lab-scale experiments.

### 3.4. Challenges and Perspectives Regarding PHA Production by Mixed Microbial Consortia

PHA process brought to an industrial scale using pure substrates and strains has broadly surpassed cell densities of 150 g/L with PHA contents up to 90% and productivities in the range from 1 to 3 g PHA/L h [4]. Comparable productivities, of up to 1.2 g PHA/L h, combined with high PHA yields, up to 0.8 Cmol PHA/Cmol S, have already been attained from MMCs using synthetic substrates [137]. Likewise, PHA contents up to 90% of the CDW have been reported [109]. Thus, MMC have proven to be able to achieve comparable results to pure cultures in synthetic media. Nevertheless, PHA productivities could be further increased if higher cell densities were obtained, a parameter that is usually below 10 g/L in MMC. In pure cultures, the PHA accumulation phase is preceded by a biomass growth phase in order to achieve high biomass densities [154]. In the accumulation phase, the feeding strategy is modified accordingly (usually limiting the nitrogen) to obtain a high PHA content. Nevertheless, in processes for PHA production from MMC, the biomass generation step is also the enrichment step. Thus, two objectives, which might not have the same optimal conditions, are combined in the same process unit. A future direction could be to test if adopting a microbial biomass generation step—leading to higher cell densities before the PHA accumulation—could maintain the high PHA content of the cells in MMC.

In order to achieve a sustainable production of PHA, both in economic and environmental terms, high productivities should also be obtained in waste substrates. In the current state of art, neither pure strains nor MMC have achieved high productivities when waste streams are used as substrates [83,97,155]. Thus, the challenge is common.

One of the main issues that compromises productivity when using waste streams is their diluted nature [155,156]. This applies for the case of MMC where an anaerobic fermentation step is usually performed in order to convert sugars to fatty acids. However, it is also the case with other industrial wastes used both in pure and mixed cultures, such as whey or lignocellulosic biomass, that requires pre-processing to release its sugars [83,155]. When these effluents are provided as feed in fed-batch PHA accumulations, they provoke substantial increases in the reactor operating volume, thus reducing the productivity.

A promising way to obtain high cell densities and productivities would be the use of cell recycling systems coupled to fed-batch processes [156]. This strategy has been scarcely applied to PHA production until now, with only one report using an MMC [139] and one study in pure strains [157]. The latter obtained cell densities up to 200 g/L with a productivity of 4.6 g PHA/L/h by using external cross-flow membranes to recirculate cells into the fed-batch reactor using *C. necator* [157]. Thus, the strategy seems to offer good opportunities to increase the cell density and PHA concentration. Likewise, reactor designs preventing the cells from escaping the system, while allowing supernatant removal, could result in high cell densities and reduced reactor volumes.

With the same scope, other research groups have proposed influent concentration. Although evaporation has been suggested [157], other less energy intensive methods such as forward osmosis membranes could more likely be applied [158,159]. A very interesting approach was recently published where forward osmosis Aquaporin^®^ membranes, mimicking biological protein channels, were suggested to concentrate fermentation effluents from glycerol and wheat straw [160]. The novelty lied in the fact that the concentrated feedstock could be used as the water draw solution with the diluted fermentation effluent being the water feed solution. This enabled the recirculation of water from the effluent to the influent in an energy efficient way since the process was based solely on the use of forward osmosis membranes without the need of the costly regeneration of the draw solution (usually performed by applying the energy intensive reverse osmosis). Integration of such systems in the PHA production process could also enhance its productivity.

## 4. PHA Recovery

**Figure 2:**
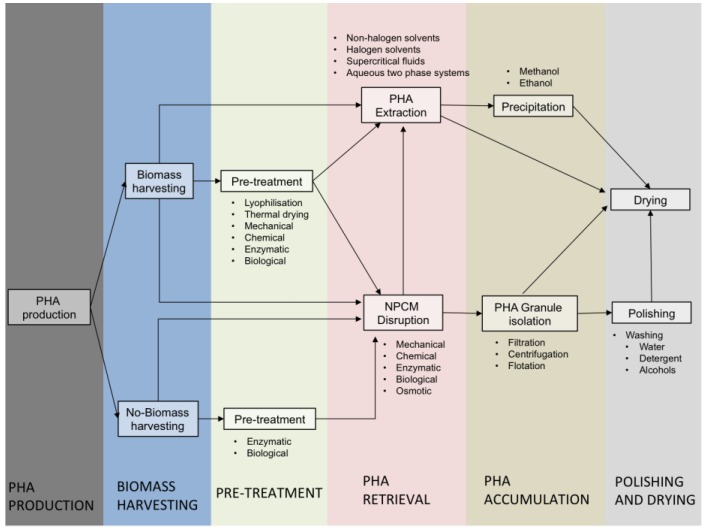
PHA recovery strategies.

The development of new strategies and methodologies for PHA recovery is one of the main factors associated with the feasibility of a PHA production bio-refinery using microbial mixed fermentation. As demonstrated in Figure 2, PHA recovery uses a variation of different techniques. However, PHA purification generally requires five steps: biomass-harvesting, pre-treatment, PHA recovery, PHA accumulation, polishing, and drying. Biomass harvesting is the concentration of biomass using techniques such as filtration or centrifugation. As PHAs are intracellular polymers, it is necessary to concentrate the biomass prior PHA recovery. Nevertheless, some researchers have evaluated PHA recovery without biomass harvesting to facilitate process scale-up and to reduce costs. Pre-treatment’s main objective is to facilitate PHA retrieval from the microbial biomass; these techniques include drying techniques (lyophilization and thermal drying), grinding, chemical and biochemical pre-treatments, etc. The pre-treatment step can combine two or more methods. PHA retrieval phase utilizes two principal methods: PHA solubilization and the disruption of non-PHA cell mass (NPCM). In some cases, NPCM disruption precedes a PHA solubilization step. The PHA accumulation step is dependent on the retrieval technique utilized. In PHA solubilization, the PHA is concentrated by using alcohols precipitation. On the other hand, in NPCM disruption, recovery is performed by collecting the PHA granules. As final steps, recovered PHAs can be polished by removing residues, from the previous steps, or can be dried; depending on the separation steps utilized. As of 2013, two reviews specialized in PHA recovery were published [161,162]. This section primarily focuses in the most recent developments in pre-treatments and PHA retrieval steps; additionally, this section describes a more industrial opinion on the PHA recovery methods.

### 4.1. Pre-Treatments

Pre-treatments are chemical, physical, or biological methods employed to facilitate the retrieval of PHA. These methods focus on weakening the cell structure that protects and surrounds the PHA granules. After biomass harvesting, drying is the traditional pre-treatment used in PHA recovery, which includes heat drying and lyophilization; the latter is the most employed pre-treatment in PHA recovery processes. This technique removes the majority of water molecules in the biomass facilitating the posterior PHA extraction. Although lyophilization has interesting features, it has economic and technical difficulties that reduce its future application in an industrial PHA recovery process. In recent years, these industrial difficulties increased the interest in PHA recovery from wet biomass instead of dry biomass [163,164,165]. Lyophilization can be a unique pre-treatment or it can precede a further pre-treatment. Lyophilization has preceded thermal, mechanical, and chemical pre-treatments (see Table 5). All these treatments included a further retrieval technique associated with solvent extraction [166,167]. Samori et al. [166], described chemical pre-treatment with NaClO as the best pre-treatment for PHA recovery compared with thermal and mechanical pre-treatments; however, this pre-treatment generated an important reduction in the molecular weight of the polymer.

Pre-treatments without lyophilization consisted of chemical and physical methods, with chemical methods including sodium chloride (NaCl) and sodium hypochlorite (NaClO). Anis et al. [163], employed NaCl as a pre-treatment for a NaClO digestion. The additional pre-treatment step generated an increment in purity and yield. NaCl pre-treatment modifies the osmotic conditions in the medium leading the cells to dehydrate and shrink, this destabilizes the cell membranes facilitating the PHA granules’ liberation. Physical methods include high temperature and ultrasonication methods both methods anteceded NPCM digestion methods. Neves and Muller [168] evaluated three temperatures (121, 100, and 80 °C) during 15 min as pre-treatment conditions. The 121 °C treatment achieved the best results; whereas, 100 and 80 °C treatments recovered significantly lower amounts of PHA. The heat treatment improves the PHA removal by denaturizing proteins, DNA and RNA, destabilizing the microbial cell wall and inactivating the PHB depolymerase [168]. Ultrasonication employs sound waves to create disruption in the cell wall and open the cytosolic material to the aqueous medium. Leong et al. [164] utilized ultrasonication prior an aqueous two-phase extraction. The advantages of this method are the lack of any previous cell harvesting method and the fast pace in which is performed.

Pre-treatments can increase the recovery and purity of the PHA extracted from a fermentation broth. However, their implementation in a PHA industrial recovery process needs to be evaluated using economic and technical analysis. The yield and purity increment should counterbalance with the additional cost associated with the introduction of this step in the purification line. Additionally, it is important to remark that even though several pre-treatments can be used, not all of them are suitable for industrial applications. Industrially suitable pre-treatments require the use of wet biomass or unharvested biomass in order to reduce the number of purification steps and the costs associated with the purification process. Lyophilization use should concentrate on PHA chemical analyses or be replaced with more suitable drying techniques for process scale up. The selection of a pre-treatment is dependent of the bacterial strain, fermentation broth characteristics, and further PHA application; therefore, each PHA process needs to be analyzed individually from an economic, environmental, and technical point of view.

### 4.2. Retrieval Techniques

#### 4.2.1. Non-PHA Cell Mass (NPCM) Disruption

In recent years, the use of NPCM disruption as a tool for retrieving PHA from bacterial biomass has increased. This increment is associated with the necessity of environmental and safe options to replace the use of halogenated solvents used in the traditional PHA extraction methods. Additionally, some of the NPCM techniques for the PHA extraction have used wet biomass or unharvested biomass, which is an advance through the reduction of purification steps and costs. This review grouped the novel NPCM disruption techniques into chemical, enzymatic, and biological disruption.

##### Chemical Disruption

NPCM chemical disruption includes methods that utilize chemical compounds to disrupt bacterial cell wall and the denaturalization or degradation of cytosolic material. The three principal methods for the chemical disruption are sodium hydroxide (NaOH), sodium hypochlorite (NaClO), and sodium dodecyl sulphate (SDS). Additional chemical treatments include water and acid treatments. NaOH treatment destabilizes the cell wall by reacting with the lipid layer in a saponification reaction and increases the cell membrane permeability [163]. NaOH treatment has obtained high recovery and purification percentage using pre-treated and unpretreated biomass (see Table 6). However, pre-treated biomass aided the NaOH treatments to achieve improvements in purity and recovery [163,169]. The combination of NaCl pre-treatment and NaOH digestion increased the purity and recovery of NaOH treatment by approximately 10% [163]. Similarly, lyophilization and freezing helped to increase the PHA purity using NaOH; however, these pre-treatments did not increase the recovery percentage [169]. López-Abelairas et al. [170] described a recovery reduction in treatments with high biomass and NaOH concentrations. Biomass concentration above 2.5% created a constant reduction in recovery and purity; however, biomass concentrations of 7.5% and 10% yielded the greatest recovery reductions. NaOH concentrations over 0.5 N affected recovery, although, the purity percentage at high percentage was constant. Likewise, Villano et al. [171] achieved recovery between 80–87% utilizing high NaOH (1 M), high biomass concentration (6:1 biomass:chemical solution) and extended extraction times (6–24 h). However, the purity achieved by this treatment was below 60%.

Sodium hypochlorite (NaClO) has confirmed its positive aspects as an NCMP disruption treatment [161,162]. Therefore, in recent years, the assessments of this treatment advanced to larger scales or continuous processes [171,172]. A continuous sequential process for PHA production and recovery obtained high polymer recovery (100%) and purity (98%). This continuous process contains three steps: a production step using microbial mixed culture, a PHA accumulation step, and a PHA extraction step using NaClO (5%) disruption for 24 h. This approach produced 1.43 g PHA/L·d and was stable for four months [171].

Another chemical disruption treatment is sodium dodecyl sulphate (SDS). This surfactant is a well-known detergent used in the recovery of genetic material. SDS treatment obtained recovery and purity values comparable with other chemical disruption techniques; moreover, this disruption technique obtained similar retrieval with and without biomass pre-treatment [169,173]. The amount of SDS varied between 0.025% and 0.2%, higher concentrations of SDS generated higher purity as result of SDS micelles formation. High micelles production is also associated with the solubilization of PHA granules generating a reduction in the recovery yield. SDS has complemented NaOH disruption, this combination exhibited superior levels of purity, especially in the removal of hydrophobic impurities [169].

Besides the previous treatments, other authors have described water and acid disruption methods as effective treatments for NPCM disruption. Distilled water achieved high purity (94%) and recovery (98%) percentages; however, the process needed 18 h and lyophilized biomass to reach these high percentages. The process duration improved by adding SDS (0.1%) into the mixture [173]. In contrast, distilled water disruption treatment with wet biomass obtained recovery (80%) and purity (58%) percentages lower than lyophilized biomass. Mohammadi et al. [174], described a higher purity and recovery yield using distilled water disruption with recombinant bacteria instead of wild bacteria. Recombinant bacterial cell wall is thinner than in wild type bacteria, which facilitates the cell wall breaking by osmotic pressure. Acid treatments have demonstrated their capability to disrupt NPCM; López-Abelairas et al. [170] described a recovery and purity percentage using a sulphuric acid solution (0.64 M) similar to alkaline treatments (NaOH, NaClO). They selected acid disruption as the best recovery method focused in operational and environmental factors. The authors chose acid disruption because this process had lower cost, environmental impact (greenhouse gas emissions), and polymer degradation than alkaline treatments [170].

Chemical disruption treatments are a significant option in PHA recovery since they present environmental and economic advantages over the traditional PHA extraction using halogenated solvents. Environmentally, chemical disruption avoids the use of toxic solvents such as chloroform. Economic advantages include liquid current recycling, the use of wet biomass, and the reagent cost. The principal drawback for chemical disruption has been polymer degradation; however, the use of mixtures of chemicals and process optimization has reduced polymer degradation. The selection of a chemical disruption method for PHA recovery from mixed microbial cultures needs an all-around evaluation of technical, economic, and environmental factors that consider the positive and negative effects and how they can affect the feasibility of a PHA bio-refinery plant.

##### Enzymatic NPCM Disruption

Enzymatic NPCM disruption utilizes purified enzymes or crude extracts to disrupt the bacterial cell wall. Proteases are the principal enzymatic activities employed in enzymatic disruption; however, other types of enzymes or enzymatic cocktail have effectively degraded NCMP. Enzymatic disruption advantages include their low energy requirements, aqueous recovery, and low capital investment; in contrast, the enzymes production cost is the principal disadvantage for industrial implementation [175]. Gutt et al. [176], evaluated the recovery of P3HBHV from *Cupriavidus necator* by several methods including enzymatic disruption. Simple enzymatic treatment (lysozyme) obtained low recovery and purity (Table 6). The authors attributed these low percentages to the absence of additional chemical or mechanical treatments, which have proved necessary for achieving high recovery and purity. Martino et al. [177] evaluated enzymatic disruption using simultaneously enzymatic (Alcalase) and chemical treatments (SDS and EDTA). SDS and EDTA contributed to cell wall and membrane lysis whereas Alcalase solubilized the cytosolic material. This enzymatic/chemical digestion treatment eliminated the requirement of heat pre-treatment used in previous enzymatic disruption researchers [161,162]. Kachrimanidou et al. [175], developed a novel enzymatic disruption method by using crude enzymes from solid-state fermentation of *Aspergillus awamori.* This method achieved good recovery (98%) and purity (97%) without using additional chemicals; however, it required heat and lyophilization as pre-treatments. Approaches focused in the reduction of enzyme costs are necessary to facilitate the industrial application of enzymatic disruption including the use of immobilized enzymes, integration of enzymes production as part of a PHA biorefinery, and genetically engineering enzymes.

##### Biological NPCM Disruption

Biological disruption utilizes biological agents (virus) or organisms to liberate PHA from bacterial cells. The first biological disruption technique used viral particles to break bacterial cells. Bacteriophages were included in bacterial lines to utilize the viruses’ lytic cycle to liberate PHA granules. When the lytic cycle is completed, the virus escapes from the host cell by breaking down the cell wall; this breaking down also liberates the PHA particles allowing their recovery [162]. In recent years, biological disruption methods included bacteria predators, rats, and mealworms [178,179,180].

Martinez et al. [181], proposed the use of obligate predatory bacteria *Bdellovibrio bacteriovorus* as an innovative cell lytic agent suitable to recover intracellular bioproducts such as PHA. *B. bacteriovorus* achieved a PHA recovery of 60%, the recovery percentage obtained was attributed to PhaZ depolymerase activity which hydrolyses PHA and expresses during all the stages of *B. bacteriovorus*’s life cycle [181]. To improve the use of *B. bacteriovorus* in PHA recovery, Martinez et al. [180], developed *B. bacteriovorus* mutant strains, one with an inactive medium-chain-length PHA depolymerase (*B. bacteriovorus* Bd3709) and another with inactive short-chain-length-PHA depolymerase (*B. bacteriovorus* Bd2637). *B. bacteriovorus* Bd3709 increased PHA recovery from 60% to 80% when predating *Pseudomonas putida*, whereas, *B. bacteriovorus* Bd2637 increased PHB recovery from 48% to 63% when predating PHB-accumulating *E. coli* ML35 [180]. Biological recovery using *B. bacteriovorus* has advantages such as avoiding the use of cell harvesting and pre-treatments. However, it has disadvantages such as the use of organic solvent steps, processing time, and low recovery. In the future, this biological method can be complemented with other recovery treatments to avoid solvents’ usage and to increase PHA recovery.

In recent years, authors have used complex organisms’ digestive system as a NPCM disruption technique. In these treatments, different organisms were fed with PHA-rich bacteria; afterwards, PHA granules were recovered from these organism’s feces. This process selectively digested the NPCM without reducing the PHA molecular weight. Kunasundari et al. [182], fed Sprague Dawley rats with lyophilized cells of *Cupriavidus necator* in a single cell protein diet during several days. The fecal pellets were whitish and rich in PHA (82–97%). The authors also demonstrated the safety and tolerability of the Sprague Dawley rats to a *Cupriavidus necator* diet*.* Kunasundari et al. [179], studied the purification of the Sprague Dawley PHA-rich fecal pellets using water and surfactants. The use of SDS 2% as a further purification step increased the purity of the PHA biological recovered at levels similar to solvent extraction. Similar to Sprague Dawley rats, Murugan et al. [180] fed lyophilized bacteria to mealworms and recovered PHA granules from their fecal pellets. The PHA granules had an 89% purity when washed with water and reached almost 100% purity when treated with SDS. The authors reported higher protein content in mealworms fed with *C. necator* cells than mealworms fed with oats. Mealworms fed with *C. necator* can be an alternative protein source in aquaculture and poultry diets. Biological NPCM disruption is an alternative to other disruption methods; it does not require expensive instrumentation, solvents, or strong chemicals, and the organisms doing the PHA recovery can be a marketable product too. However, the biological recovery process takes longer than any other recovery process and needs biomass pre-treatment. Depending on the final use, PHA biological recovery can be an integrated process for renewable production of feed, food, and materials.

#### 4.2.2. PHA Extraction

As PHA are produced inside the cellular biomass, in order to be retrieved they have to be separated from the non-PHA cell mass (NPCM). The simplest, least destructive to biopolymer and most direct way for PHA is to be extracted from the biomass; significant quantities of hazardous solvents and energy input are required for this, creating a potential counterbalance to sustainability and economics towards commercialization [184].

Several studies have proposed various solvent extraction methods for PHA recovery that improve parameters such as yield, purity, and cost of extraction, while at the same time maintaining the physicochemical properties of the biopolymer [185].

##### Non-Halogenated Solvents

Although several types of extraction systems exist for the production of biopolymers, the majority of extraction methods for PHA still involve the use of organic solvents in which the polymer is soluble. Fei et al. [186], aimed to develop an effective and environmentally-friendly solvent system so as to extract PHB from bacterial biomass. In order to accomplish that, they used a solvent mixture of acetone/ethanol/propylene carbonate (A/E/P, 1:1:1 v/v/v) for extracting PHB from *Cupriavidus necator*. When the A/E/P mixture was used at high temperature, it could recover 92% pure PHB with 85% yield from dry biomass, and 90% purity with 83% PHB yield from wet biomass. Additionally, if hexane was added, it could further enhance the purity and recovery quantities of PHB.

Bacterial PHA could be used for medical applications due to its biocompatibility. However, using inappropriate solvents or techniques during extraction of PHA from bacterial biomass could result in contamination by pyrogenic compounds (e.g., lipopolysaccharides), which eventually leads to rejection of the material for medical use. This problem could be overcome by using a temperature-controlled method for the recovery of poly(3-hydroxyoctanoate-co-3-hydroxyhexanoate) from *Pseudomonas putida* GPo1. Non-chlorinated solvents were found to be the optimal solvents for such tests and, specifically, n-hexane and 2-propanol. The purity reached more than 97% (w/w) and the endotoxicity between 10–15 EU/g PHA. Further re-dissolution in 2-propanol at 45 °C and precipitation at 10 °C resulted in a purity of nearly 100% (w/w) and endotoxicity equal to 2 EU/g PHA [187].

Another approach, however, is that the use of aqueous solvents could benefit the integration into a biorefinery scheme. A study aimed at connecting the exploitation of a raw biowaste, such as used cooking oil (UCO), and producing a desired final product (i.e., amorphous granules of PHA) based on aqueous solvents in a way that will prove the effective reliability of an overall biotechnological approach. Used cooking oil was utilized as the only carbon source for the production of PHB by cultivating *Cupriavidus necator* DSM 428 in batch reactors. The PHB granules were extracted from the biomass using sodium dodecyl sulfate (SDS), ethylenediaminetetraacetic acid (EDTA), and the enzyme Alcalase in an aqueous medium. The PHB granule recovery reached more than 90% and highly pure amorphous polymer was finally obtained [177].

A different alternative to the use of halogen-free and environmental-friendly methods implemented the use of water and ethanol for the recovery of PHA from recombinant *Cupriavidus necator*, in comparison to the well-established chloroform extraction method. Comparing the results obtained from experiments under different incubation times (1, 3, and 5 h) and temperatures (4 and 30 °C) showed that the optimized halogen-free method produced a PHA with 81% purity and 96% recovery yield, whereas the chloroform extraction system resulted in a highly pure PHA with 95% recovery yield. This method could potentially be developed as an alternative and more environmentally-friendly method for industrial application [188].

##### Aqueous Two-Phase Extraction Systems (ATPS)

In addition to the conventional isolation and purification methods, such as solvent extraction, aqueous two-phase systems (ATPS) have many advantages and important characteristics that attract the attention of researchers and industries. The main advantages are that because ATPS comprise of high water content (70–90% w/w), thus they provide a beneficial environment for separation of sensitive biomaterials. Also, the materials that form the different phases/layers of ATPS are, in principle, safe and environmentally-friendly compared to conventional solvent extraction methods; additionally, an intricate ability for high capacity processing which leads to reduced purification steps; finally, large-scale purification using ATPS can be easily and reliably predicted from laboratory experimental data. ATPS is a feasible solution for industrial demand of cost-effective and highly efficient large-scale bioseparation technologies with short processing times [189,190].

Regarding PHA retrieval methods, one interesting approach is the use of aqueous two-phase systems with the aim of enhancing the accumulation of PHA in one phase using environmentally-friendly layer-forming constituents. This method offers advantages of supporting a beneficial environment for bioseparation, capability of handling high operating capacity, and reducing downstream processing volume, thus proving extractive bioconversion via ATPS can be an optimum solution. Leong et al. [189], examined the effect of pH and salts’ addition in *Ralstonia eutropha* H16 cultures, using an ATPS system as a mechanism for PHA extraction. The optimum result obtained in this study was a PHA concentration of 0.139 g/L (purification factor: 1.2–1.63) and recovery yield 55–65% using ATPS of polyethylene glycol (PEG) 8000/sodium sulphate adjusted to pH 6 and the addition of 0.5 M NaCl.

In another study using ATPS, the thermal separation of the phases and how it affected the PHA extraction was studied. The most important ATPS parameters (type and concentration of thermoseparating polymer, salt addition, feedstock load, and thermoseparating temperature) were optimized in order to achieve high PHA retrieval from the bacterial lysate. By taking advantage of the properties of the thermo-responsive polymer (whose solubility decreases in its aqueous solution as the temperature increases), cloud point extraction (CPE) is an ATPS technique that offers the capability to its phase-forming component to be reused. Extraction of PHA from *Cupriavidus necator* H16 via CPE was investigated. The best conditions for PHA extraction (recovery yield of 94.8% and purification factor 1.42) were reached under the following conditions: 20% w/w ethylene oxide-propylene oxide (EOPO), 10 mM NaCl addition, and a thermoseparating temperature of 60 °C with crude feedstock limit of 37.5% w/w. Another benefit of this process is the ability to recycle and reuse EOPO 3900 at least twice, achieving a satisfying yield and purification factor [164].

## 5. Conclusions

Wide production, commercialization, and thus application of PHAs as a biodegradable alternative to conventional plastics is still limited due to high production cost. Bioprocess technologies are still being developed while bacterial resources are still being explored.

Pure cultures are constantly investigated for their potential to valorize waste byproducts as a low-cost feedstock. The ability of certain bacteria to directly utilize lignocellulosic biomass as the carbon source for PHA production is a huge bioprocess advantage. In this case, the need for chemicals, energy, and labor is minimized. PHA production can be also used as a tool for the bioremediation of oil contaminated sites, as bacterial strains can degrade environmental pollutants, minimizing their toxicity and environmental impact. Moreover, halophilic PHA producers combine a series of advantages, such as growth on seawater and possibility of continuous processes under non-sterile conditions. Those features will significantly contribute to the PHA cost reduction and minimize the requirements for fresh water. Synthetic biology tools are expected to aid in the enhancement of PHA production efficiency, simplify downstream process, and regulate PHA composition providing customized materials for specific applications. However, robust strains are yet to be developed.

Efforts have been also made towards the PHA production using mixed microbial cultures (MMC). MMC-based processes, apart from offering a reduction in the operational costs and the possibility to adapt to a wider range of waste substrates, they could be integrated in current wastewater treatment plants. Recent developments regarding different enrichment strategies and the PHA production step offer new opportunities to make the PHA production more feasible. Cell densities and derived productivities attained with MMC are the main current bottleneck.

The development of economic and simple downstream processes is crucial for the recovery of PHAs. Methods based on the utilization of environmentally friendlier techniques are constantly being investigated, with enzymatic methods advancing the bio-based profile of the process. Reduction of large amounts of chemicals, used per cell dry mass, is going to benefit the economics of the process as well as society and the environment.

## Figures and Tables

**Table 1:** Pilot and industrial scale PHA manufacturers currently active worldwide.

Name of Company	Product (Trademark)	Substrate	Biocatalyst	Production Capacity
Biomatera, Canada	PHA resins (Biomatera)	Renewable raw materials	Non-pathogenic, non-transgenic bacteria isolated from soil	
Biomer, Germany	PHB pellets (Biomer^®^)	Sugar (sucrose)		
Bio-On Srl., Italy	PHB, PHBV spheres (minerv^®^-PHA)	Sugar beets	*Cupriavidus necator*	10,000 t/a
BluePHA, China	Customized PHBVHHx, PHV, P3HP3HB, P3HP4HB, P3HP, P4HB synthesis		Development of microbial strains via synthetic biology	
Danimer Scientific, USA	mcl-PHA (Nodax^®^ PHA)	Cold pressed canola oil		
Kaneka Corporation, Japan	PHB-PHHx (AONILEX^®^)	Plant oils		3500 t/a
Newlight Technologies LLC, USA	PHA resins (AirCarbon^TM^)	Oxygen from air and carbon from captured methane emissions	Newlight’s 9X biocatalyst	
PHB Industrial S.A., Brazil	PHB, PHBV (BIOCYCLE^®^)	Saccharose	*Alcaligenes* sp.	3000 t/a
PolyFerm, Canada	mcl-PHA (VersaMer^TM^ PHA)	Sugars, vegetable oils	Naturally selected microorganisms	
Shenzhen Ecomann Biotechnology Co. Ltd., China	PHA pellets, resins, microbeads (AmBio^®^)	Sugar or glucose		5000 t/a
SIRIM Bioplastics Pilot Plant, Malaysia	Various types of PHA	Palm oil mill effluent (POME), crude palm kernel oil		2000 t/a
TianAn Biologic Materials Co. Ltd., China	PHB, PHBV (ENMAT^TM^)	Dextrose deriving from corn of cassava grown in China	*Ralstonia eutropha*	10,000 t/a, 50,000 t/a by 2020
Tianjin GreenBio Material Co., China	P (3, 4HB) films, pellets/foam pellets (Sogreen^®^)	Sugar		10,000 t/a

PHB, P3HB: poly(3-hydroxybutyrate); PHBV: poly(3-hydroxybutyrate-co-3-hydroxyvalerate); PHBVHHx: poly(3-hydroxybutyrate-co-3-hydroxyvalerate-co-3-hydroxyhexanoate); PHV: poly-3-hydroxyvalerate; P3HP3HB: poly(3-hydroxypropionate-co-3-hydroxybutyrate); P3HP4HB: poly(3-hydroxypropionate-co-4-hydroxybutyrate); P3HP: poly(3-hydroxypropionate); P4HB: poly(4-hydroxybutyrate); mcl-PHA: medium-chain length PHA; P(3,4HB): poly(3-hydroxybutyrate-co-4-hydroxybutyrate).

**Table 2:** Characteristic parameters describing PHA production from different types of bacteria.

Strain	Carbon Source	PHA	Cultivation Mode	DCW (g L^−1^)	PHA (g L^−1^)	PHA (%)	*Y*_P/S_	Ref.
Lignocellulose Degraders								
*S. degradans*	Glucose	PHB				17.2		[20]
	Glucose	PHB	Fed-batch	12.7	2.7	21.4	0.17	[21]
	Starch		Fed-batch	11.7	2.0	17.5	0.14	
	Glucose	PHB	Flask	2.1	0.46	22.4		[23]
	Cellobiose		Flask	2.0	0.42	20.8		
	α-Cellulose		Flask	1.2	0.14	11.8		
	Avicel		Flask	1.0	0.15	14.6		
	Sigmacell		Flask	1.0	0.14	13.7		
	CMC		Flask	1.1	0.14	12.7		
	Glucose		Batch	1.6	0.40	25.3		
	Glucose		Fed-batch	4.2	2.20	52.8		
	Avicel		Fed-batch	2.1	0.40	19.2		
	Agarose	PHB	One-step batch		0.24	18.1		[24]
	Xylan		One-step batch		0.20	22.7		
	Agarose		Two-step batch		0.31	18.4		
	Xylan		Two-step batch		0.24	15.3		
*Co-culture* of *S. degradans* and *B. cerues*	Agarose	PHB	One-step batch		0.29	19.7		[24]
	Xylan		One-step batch		0.27	34.5		
	Agarose		Two-step batch		0.23	15.3		
	Xylan		Two-step batch		0.33	30.2		
*C. taiwanensis*	Propionate + Glc ^a^	PHBV (88–12) ^f^	Flask	2.0	1.04	52		[22]
	Valerate + Glc ^a^	PHBV (49–51)		1.0	0.51	51		
	Hexanoate + Glc ^a^	PHBHHx ^c^		2.7	1.67	62		
	Hexanoate + Glc ^a^ + AA ^b^	PHBHHx ^d^		1.2	0.56	47		
	Heptanoate + Glc ^a^	PHBV (65–35)		1.7	0.56	33		
	Heptanoate + Glc ^a^ + AA	PHBV (15–85)		0.3	0.05	17		
	Octanoate + Glc ^a^	PHB		0.4	0.05	13		
	Cassava starch + Val ^e^	PHBV (87–13)		2.8	1.88	67		
	Corn starch + Val ^e^	PHBV (80–10)		3.3	2.14	65		
	Potato + Val ^e^	PHBV (80–10)		2.6	1.43	55		
	Sweet potato + Val ^e^	PHBV (80–10)		1.6	0.83	52		
	Wheat starch + Val ^e^	PHBV (80–10)		4.1	1.72	42		
Polyhydroxyalkanoates and Bioremediation								
*P. putida* F1	Benzene	mcl-PHA	Flask	0.34	0.05	14		[41]
	Toluene			0.72	0.16	22		
	Ethylbenzene			0.67	0.10	15		
*P. putida* mt-2	Toluene	mcl-PHA	Flask	0.37	0.08	22		[41]
	*p*-Xylene			0.53	0.14	26		
*P. putida* CA-3	Styrene	mcl-PHA	Flask	0.79	0.26	33		[41]
*P. fluva* TY16	Benzene	mcl-PHA	Continuous feeding	2.54		19	0.03	[42]
	Toluene			3.87		59	0.11	
	Ethylbenzene			2.80		29	0.04	
*P. putida* CA-3	Styrene pyrolysis oil	mcl-PHA	Flask	2.80	1.60	57	0.10	[44]
*Sphingobacterium* sp. ATM	Orange 3R dye	PHA	Flask		3.48	65		[38]
*B. odysseyi* SUK3					2.10	61		
*P. desmolyticim* NCIM 2112					1.12	52		
Halophiles								
*H. mediterranei* DSM 1411	25% pre-treated vinasse	PHBV (86–14)	Flask		19.7	70	0.87	[55]
	Stillage	PHBV (85–15)			16.4	71	0.35	[56]
	Hydrolyzed cheese whey	PHBV (98.5–1.5)	Batch	7.54		54	0.78	[57]
	15% v/v olive mill wastewater	PHBV (94-6)	Flask		0.2	43		[58]
*Halomonas* TD01	Glucose salt medium	PHA	Continuous two-fermentor			65	0.51	[59]
*Halomonas campaniensis* LS21	Mixed substrates (mostly comprised of kitchen waste)	PHB	Continuous pH-stat			26		[60]
*B. megaterium* H16	Glucose salt medium	PHB	Flask			39		[61]
*B. megaterium* uyuni S29	Glucose salt medium	PHB	Flask	5.42	2.22	41	0.13	[63]

^a^ Mixtures consisting of 0.1% fatty acid and 1.5% gluconate; ^b^ 2mM acrylic acid; ^c^ 99.5% HB, 0.5% HHx; ^d^ 98.5% HB, 1.5% HHx; ^e^ Mixtures consisting of 1.5% starch type + 0.05% Valerate; ^f^ PHBV (%HB–%HV).

**Table 3:** Summary on the main characteristics of the enrichment techniques applied for MMCs.

**Anaerobic-Aerobic Enrichment (AN/AE) (Section 3.1.1)**			
		**Feast phase**	**Famine phase**
**Aeration**		No	Yes
**e^−^ acceptor**		-- (PHA)	Oxygen
**Energy source**		Glycogen/polyphosphate	Oxidation of PHA
**Carbon source**		External substrate	PHA
**Driving force for PHA accumulation**		• Lack of electron acceptor *	
		• Transient presence of substrate ***	
**Aerobic Dynamic Feeding (ADF) (Section 3.1.2 and Section 3.1.3)**			
*Classical Aerobic Dynamic Feeding (Section 3.1.2)*			
		**Feast phase**	**Famine phase**
**Aeration**		Yes	Yes
**e^−^ acceptor**		Oxygen	Oxygen
**Energy source**		Oxidation of substrate	Oxidation of PHA
**Carbon source**		External substrate	PHA
**Nitrogen availability**		Yes **	Yes **
**Driving force for PHA accumulation**		• Transient presence of substrate ***	
*Aerobic Dynamic Feeding (ADF) with Intermediate Settling Phase (Section 3.1.3)*			
		**Feast phase**	**Famine phase**
**Aeration**		Yes	Yes
**e^−^ acceptor**		Oxygen	Oxygen
**Energy source**		Oxidation of substrate	Oxidation of PHA
**Carbon source**		External substrate	PHA
**Nitrogen availability**		Yes	Yes
**Driving force for PHA accumulation**		• Transient presence of substrate ***	
		• Higher settling capacity of PHA rich cells	
		• Elimination of residual COD after feast phase prevents growth of non-PHA accumulating bacteria	
*Aerobic Dynamic Feeding (ADF) with Nitrogen Limitation in the Feast-Phase (Section 3.1.3)*			
		**Feast phase**	**Famine phase**
**Aeration**		Yes	Yes
**e^−^ acceptor**		Oxygen	Oxygen
**Energy source**		Oxidation of substrate	Oxidation of PHA
**Carbon source**		External substrate	PHA
**Nitrogen availability**		No	Yes
**Driving force for PHA accumulation**		• Transient presence of substrate ***	
		• Nitrogen limitation during the feast phase	
**Photosynthetic Enrichment (Section 3.1.5)**			
*Photosynthetic Enrichments—Illuminated SBR*			
		**Feast phase**	**Famine phase**
**Aeration**		No	No
**e^−^ acceptor**		-- (PHA)	Oxygen produced by algae
**Energy source**		Light	Oxidation of PHA + Light
**Carbon source**		External substrate	PHA
**Driving force for PHA accumulation**		• Lack of external electron acceptor with presence of light	
		• Transient presence of substrate ***	
*Photosynthetic Enrichment—Dark Feast Phase*			
		**Feast phase**	**Famine phase**
**Aeration**		No	No
**e^−^ acceptor**		-- (PHA)	Oxygen produced by algae
**Energy source**		Glycogen	Oxidation of PHA + Light
**Carbon source**		External substrate	PHA
**Driving force for PHA accumulation**		• Lack of external electron acceptor with presence of light	
		• Transient presence of substrate ***	
*Photosynthetic Enrichment—Permanent Feast Phase*			
		**Feast phase**	**Famine phase**
**Aeration**		No	No famine phase
**e^−^ acceptor**		-- (PHA)	
**Energy source**		Light	
**Carbon source**		External substrate	
**Driving force for PHA accumulation**		• Lack of external electron acceptor with presence of light	
**Aerobic-Anoxic Enrichment (Section 3.1.6)**			
	**Feast phase**		**Famine phase**
**Aeration**	Yes		No
**e^−^ acceptor**	Oxygen		NO_3_ /NO_2_
**Energy source**	Oxidation of substrate		Oxidation of PHA
**Carbon source**	External substrate		PHA
**Driving force for PHA accumulation**	• Transient presence of substrate ***		
**Anoxic-Aerobic Enrichment (Section 3.1.7)**			
	**Feast phase**		**Famine phase**
**Aeration**	No		Yes
**e^−^ acceptor**	NO_3_/NO_2_		Oxygen
**Energy source**	Oxidation of substrate		Oxidation of PHA
**Carbon source**	External substrate		PHA
**Driving force for PHA accumulation**	• Transient presence of substrate ***		
**Microaerophilic Enrichment (Section 3.1.8)**			
	**Feast phase**		**Famine phase**
**Aeration**	Yes		Yes
**e^−^ acceptor**	Oxygen		Oxygen
**Energy source**	Oxidation of substrate		Oxidation of PHA
**Carbon source**	External substrate		PHA
**Driving force for PHA accumulation**	• Transient presence of substrate ***		
	• Limitation of electron acceptor		

* Even though the lack of electron acceptor is the driving force of the enrichment, this limitation is not mandatory for these cultures to produce PHA, which can also be produced aerobically. ** various C/N ratios have been applied resulting in a limitation of nitrogen in the famine or late feast phase. Nevertheless, most wide-spread configuration provides nitrogen in both phases *** Transient presence of substrate leads to the following effects in all cases mentioned in the table: Growth during famine phase consuming the PHA accumulated; Limitation of internal growth factors; Higher responsiveness of PHA producers to substrate addition.

**Table 4:** Main characteristics of PHA production in pilot-scale.

Pilot Plant, Location	Feedstock	Origin of MMC and Enrichment Strategy	*Y*_P/S_ (g/g) *	PHA % (%mol HB: %mol HV)	mg PHA/g X/h	Ref.
Nagpur, India	Pre-fermented milk and ice cream processing wastewater	Activated sludge	0.425 *	39–43		[150]
Lucun WWTP in Wuxi, China	Hydrolyzed and acidified raw excess sludge	Activated sludge/synthetic mixture of VFA, ADF feast famine with carbon limitation and inhibitor of nitrification	0.044–0.29 *		2.06–39.31	[125]
Eslöv, Sweden	Beet process water, 38% in VFA	PHA producing MMC from pre-fermented effluent of Procordia Foods		60 (85:15 HB:HV)		[126]
Brussels North WWTP (Aquiris, Belgium)	Pre-hydrolyzed and fermented WWTP sludge	Sludge fed with municipal WW under aerobic feast famine	0.25–0.38	27–38 (66–74:26–34 HB:HV)	100–140	[151]
Leeuwarden WWTP, Friesland, Netherlands	Fermented residuals from green-house tomato production	Sludge fed with municipal WW under anoxic feast/aerobic famine	0.30–0.39	34–42 (51–58:42:49 HB:HV)	28–35	[152]
Mars company, Veghel, Netherlands	Fermented wastewater from a candy bar factory	Activated sludge from a WWTP fed with the fermented wastewater under aerobic feast/famine with inhibitor of nitrification	0.30	70–76 (84:16 HB:HV)		[153]

* Yield calculated on a COD basis by using the coefficients: for HB: 1.67 g COD PHA/g PHA and for HV: 1.92 g COD PHA/g PHA.

**Table 5:** Pre-treatment techniques applied for PHA recovery.

Pre-Treatment	Further PHA Retrieval Treatment	Pre-Treatment Conditions	Purity (%)	Recovery (%)	Ref.
Sodium chloride (NaCl)	NaOH digestion	NaCl (8 g/L), 30 °C, 3 h	97.7	97.5	[163]
Ultrasonication	Aqueous two-phase extraction	Ultrasonication at 30 kHz per cycle 15 min		-	[164]
Sodium hypochlorite (NaClO)	Non-halogenated solvent extraction	NaClO (10%), 37 °C, 1 h		-	[165]
Thermal pre-treatment	Enzymatic digestion and chloroform extraction	Autoclave, 15 min 121 °C		94.1	[168]
Thermal pre-treatment ^1^	Non-halogenated solvent extraction	150 °C, 24 h		50	[166]
Ultrasonication and glass beds ^1^	Non-halogenated solvent extraction	Glass beads (0.5 mm) and Ultrasonication (10 pulses of 2 min)		50	[166]
Sodium hypochlorite ^1^	Non-halogenated solvent extraction	NaClO (5%), 100 °C, 15 min	93	82	[166]
Hot acetone ^1^	Non-halogenated solvent extraction	Acetone, 100 °C, 30 min		-	[167]

^1^ The pre-treatment included a previous lyophilization step.

**Table 6:** NPCM chemical disruption treatments.

NPCM Digestion Type	NPCM Disruption Method	Pre-Treatment	PHA Accumulation Method	Disruption Conditions	Microbial Strain	PHA Content in Biomass (%)	Purity (%)	Recovery (%)	Ref.
Chemical	NaOH	Chemical Pre-treatment	Centrifugation	NaOH (0.1 M), 30 °C, 1 h, 350 rpm	*C. necator*	68	90.8	95.3	[163]
Chemical	NaOH		Centrifugation	NaOH (0.1 M), 30 °C, 1 h, 350 rpm	*C. necator*	68	82.7	94.4	[163]
Chemical	NaOH	Lyophilization	Centrifugation	NaOH (0.1 M), 30 °C, 1 h,	*C. necator*	68	80–90	80–90	[183]
Chemical	NaOH	Lyophilization	Centrifugation	NaOH 0.05 M, 3 h, 0 rpm, 4 °C	*C. necator*	30	98.6	96.9	[174]
Chemical	NaOH	Lyophilization and milling	Centrifugation	NaOH (0.5 N), 4 h, 37 °C, 500 rpm	*C. necator*	65	93	80	[170]
Chemical	NaOH		Centrifugation	NaOH (0.2 M), 200 rpm, 30 °C, 1 h	*Mixed Culture*	62–72	87	97	[169]
Chemical	NaClO		Centrifugation	NaClO (5%) 24 h	*Mixed Culture*	46	90	~100	[171]
Chemical	NaClO	Mechanical pre-treatment	Precipitation	NaClO 13% (v/v), room temperature, 1 h.	*Ralstonia eutropha*	65.2	95.6	91.3	[172]
Chemical	NaClO	Lyophilization and Milling	Centrifugation	NaClO (13%), 37 °C, 500 rpm,4 h.	*C. necator*	65	97	82	[170]
Chemical	NaOH and SDS		Centrifugation	NaOH (0.2 M) and SDS (0.2 %), 200 rpm, 30°C, 1 h	*Mixed Culture*	62–72	99	91	[169]
Chemical	SDS		NaClO and Centrifugation	SDS (0.1%), 24 h	*H. mediterranei*	70	~100	97	[55]
Chemical	SDS		Centrifugation	SDS (0.1%), 24 h	*H. mediterranei*	71.2	~100	97	[56]
Chemical	SDS		Centrifugation	SDS (0.1%), 60 °C, 2 h	*Halomonas* sp. SK5	48	94	98	[173]
Chemical	SDS		Centrifugation	SDS (0.2 %), 200 rpm, 30 °C, 1 h	*Mixed Culture*	62–72	79	63.5	[169]
Chemical	H_2_SO_4_	Lyophilization and Milling	Chemical treatment and Centrifugation	H_2_SO_4_ (0.64 M), 6 h, 80 °C	*C. necator*	65	98	79	[170]
Chemical	Water	Lyophilization	Centrifugation	dH_2_O, 30 °C, 1 h,	*Comamonas* *sp.*	30	80.6	96	[174]
Chemical	Water	Lyophilization	Centrifugation	dH_2_O, 30 °C, 18 h	*Halomonas* sp.	48	94	98	[173]
Enzymatic	Alcalase, SDS and EDTA		Centrifugation	Alcalase (0.3 U g−1), SDS (0.3 g g−1), EDTA (0.01 g g−1). Na_2_HPO_4_ buffer, 150 rpm, 55 °C, 1 h	*C. necator*	37	94		[177]
Enzymatic	Crude extract	Heat treatment and lyophilization	Centrifugation	*Aspergillus oryzae* crude extract, Na_2_HPO_4_-citric acid buffer and 47 °C	*C. necator*	78.9	98	97	[175]
Enzymatic	Lysozyme		Centrifugation	Lysozyme solution (2 mg/mL). 1 h, 3 °C	*C. necator*	41	41	75	[176]
Biological	Mealworm (*Tenebrio molitor*)	Lyophilization	Chemical treatment, centrifugation	50 g of mealworms fed 5% of their body weight per day for 16 days.	*C. necator*	37	89%		[178]
Biological	Sprague Dawley rats	Lyophilization and grinding	Chemical treatment, centrifugation	150–200 g rats were feed 15 g/day/animal, 28 days 25 °C	*C. necator*	37	89.3	100	[179]
Biological	Sprague Dawley rats	Lyophilization	Water	150–200 g rats were feed 15 g/day/animal, 28 days 25 °C	*C. necator*	54	82–97	40–47	[182]
Biological	*Bdellovibrio bacteriovorus* HD100		Centrifugation	*P. putida* was inoculated with *B. bacteriovorus* strains 48 h. 30 °C	*P. putida*	55		60	[181]
Biological	*Bdellovibrio bacteriovorus* HD100 and Bd3709		Centrifugation	*P. putida* and *E. coli* cultures were inoculated with *B. bacteriovorus* strains 48 h. 30 °C	*P. putida*	55		80	[180]
